# Fighting inequalities in times of pandemic: The role of politicized identities and interdependent self‐construal in coping with economic threat

**DOI:** 10.1002/casp.2632

**Published:** 2022-06-13

**Authors:** Ángel del Fresno‐Díaz, Lucía Estevan‐Reina, Ángel Sánchez‐Rodríguez, Guillermo B. Willis, Soledad de Lemus

**Affiliations:** ^1^ Department of Social Psychology University of Granada Granada Spain; ^2^ Institute of Psychology Jagiellonian University Kraków Poland; ^3^ Department of Social Psychology and Anthropology University of Salamanca Salamanca Spain

**Keywords:** 99%, collective actions, economic threat, interdependent self‐construal, shared politicized identity, working class

## Abstract

During the COVID‐19 pandemic, institutions encouraged social isolation and non‐interaction with other people to prevent contagion. Still, the response to an impending economic crisis must be through the collective organization. In this set of pre‐registered studies, we analyse two possible mechanisms of coping with collective economic threats: shared social identity and interdependent self‐construction. We conducted three correlational studies during the pandemic in May–October 2020 (Study 1, *N* = 363; Study 2, *N* = 250; Study 3, *N* = 416). Results show that shared identity at two levels of politicization (i.e., working‐class and 99% identities) and interdependent self‐construal mediated the relationship between collective economic threat, intolerance towards economic inequality and collective actions to reduce it. The results highlight that the collective economic threat can reinforce the sense of community—either through the activation of a politicized collective identity, such as the working class or the 99% or through the activation of an interdependent self—which in turn can trigger greater involvement in the fight against economic inequality. Please refer to the Supplementary Material section to find this article's Community and Social Impact Statement.

“Don't personalise, collectivise!” (Reicher & Drury, 2020)

The effects of pandemics are not limited to health; they also influence the world economy and cause an exacerbation of inequalities (Adams‐Prassl, Boneva, Golin, & Rauh, 2020; Aspachs et al., 2021). For example, while the wealth of American billionaires has increased by 39%, lower classes, Latino and Black people, as well as transgender people have become increasingly vulnerable (Inequality.org., 2021). This situation is a severe injustice that infringes the rights of the most vulnerable ones limiting their access to basic resources (Oxfam, 2022). Challenging this injustice mutual aid groups emerged in many countries around the world aiming to protect the community (Ntontis et al., 2021; Stevenson, Wakefield, Felsner, Drury, & Costa, 2021). People tend to come together when facing a crisis (Bukowski, de Lemus, Rodríguez‐Bailón, Willis, & Alburquerque, 2019; Fritsche et al., 2017; Hawdon & Ryan, 2011). Also, the perception of share grievances or perceived injustice can lead people to challenge it via protests (van Stekelenburg & Klandermans, 2013; van Zomeren, Postmes, & Spears, 2008). As such, a common fate is crucial for both; the emergence of shared identities (Drury, Brown, González, & Miranda, 2016; Simon & Klandermans, 2001), and the development of a more interdependent self‐construal (Oishi & Komiya, 2017) to react to shared injustice (Drury & Reicher, 2000).

The aim of the current work was to analyse whether the collective economic threat linked to COVID‐19 is related to social identities and self‐construction which, in turn, could predict intolerance towards economic inequality and collective actions against it. Specifically, we proposed that economic collective threat might be associated with economic inequality intolerance and actions to challenge it because it is positively related to politicized collective identities (Simon & Klandermans, 2001; van Zomeren et al., 2008) (classical‐working class identity‐and emergent −99% identity‐) and interdependent self‐construal (Markus & Kitayama, 1991; Sánchez‐Rodríguez, Willis, Jetten, & Rodríguez‐Bailón, 2019).

## 1 REACTIONS TO A COLLECTIVE ECONOMIC THREAT

An uncertain context such as the pandemic increases economic injustice, promoting differences between the haves and have‐nots (Rodríguez‐Bailón, 2020). In such a context, realistic threats (e.g., scarcity in material resources and physical integrity) arise. People could perceive such threats on a personal (e.g., I could get the disease or lose my job) or on a collective level (e.g., my country will have a lack of health resources; Fritsche, Jonas, & Kessler, 2011; Fritsche & Jugert, 2017). When people feel threatened, they strive to maintain a general sense of control; when personal control is not plausible, they turn to the collective self (Stollberg, Fritsche, & Jonas, 2017). This can lead to an increase in ethnocentric attitudes, but also to supporting social change through collective actions in favour of the ingroup (Fritsche et al., 2017; Fritsche & Jugert, 2017) or in solidarity with others (Bukowski et al., 2019).

The threat is related to the identification with groups with whom we shared grievances, which in turn might lead to rejecting inequality (e.g., showing less intolerance towards inequality or being involved in collective actions and intentions; Drury & Reicher, 2000; Reicher, 1996). Among the most relevant antecedents of collective action are the perception that the context is unfair; a high group efficacy to change it, group‐based anger emotions, and a highly politized identity (van Zomeren et al., 2008). Rising economic inequality due to the pandemic may fuel all of these processes, leading to social protest. Further, the perception of a common fate may also contribute to increasing social cohesion and connection (Resta et al., 2021), promoting a more interdependent self‐construal, which could also lead them to reject inequality and promote social change. Thus, we argue that collective economic threat might promote identification with politicized groups, as well as an increase in interdependent self‐construction, as a means to reject and confront economic inequality.

## 2 POLITICIZED IDENTITIES AGAINST INEQUALITY

In order to face the shared grievances, a shared identity emerges (van Stekelenburg & Klandermans, 2017). In response to threats, social identities promote social change—especially if they are politicized (Simon & Klandermans, 2001; Stürmer & Simon, 2009; van Stekelenburg, Klandermans, & van Dijk, 2009; van Zomeren et al., 2008). In order to become politicized, people must engage as self‐conscious members in a power struggle on behalf of their group (Simon & Klandermans, 2001), and understand the need to change the structural aspects of shared grievances and injustices (Curtin, Kende, & Kende, 2016). We argue that the economic collective threat derived from the COVID‐19 pandemic might have triggered politicized identities opposing economic inequality, leading participants to express less tolerance towards inequality and more intentions to participate in collective actions against it.

From a Marxist analysis, the working‐class consciousness is what drives class struggle against the oppressor across history (e.g., feudalism, capitalism, etc.; Marx & Engels, 1848/2004). Belonging to the working class has been central to the development of political movements (e.g., labour and socialist movements, communism) and people's political and social attitudes (Easterbrook, Kuppens, & Manstead, 2020; Manstead, 2018). Thus, we proposed that identification with the working‐class can be a mobilizer to confront economic threats in the context of pandemics (cf. Žižek, 2020). However, in the last decades, the concept of the working‐class has been questioned, and some academics have redefined it (Wright, 2018). Beyond the traditional identity of a working‐class, other concepts emerge such as the precariat (Standing, 2013) or the analysis of inequality by Thomas Piketty (2013). The Occupy Wall Street movement proposed an alternative politicized identity against economic inequality: the 99%. The 99% identity is defined by the shared goal of reducing economic inequality and may be a way to maintain this shared identity but based on the reality of socio‐economic divisions within the economy and society (Stiglitz, 2012). We propose that the 99% identity can also promote a change in attitudes and support for collective actions aimed at fighting against economic inequality. Furthermore, we aim to compare the role of an old and new conceptualization of social class identification in triggering actions against inequality in the context of an economic threat.

Summing up, we argue that economic threats derived from the COVID‐19 pandemic can promote group identities that challenge the status quo, but also might trigger changes at the self‐construal level.

## 3 THE INTERDEPENDENT SELF‐CONSTRUAL

Self‐construal is a property of individuals that promotes thinking, feeling and behaving independently or interdependently as a result of the cultural contexts they inhabit (Markus & Kitayama, 1991). Independent self‐construction is defined as seeing oneself as separate from others, emphasizing one's uniqueness and self‐expression, and promoting personal goals over collective goals. In contrast, interdependent self‐construction includes seeing oneself as connected to others, fitting in with others, sacrificing one's personal goals and exercising self‐control (Markus & Kitayama, 1991).

Research on the psychology of the self, identities, and cultures proposes that the self is dynamic and malleable to the context (Markus & Hamedani, 2007; Markus & Kitayama, 2010; Stephens, Markus, & Phillips, 2014). In this line, past research has tested the relationship between economic inequality and the self‐construal. When economic inequality is high, people are more prone to perform independent self‐construction, increasing the competitive and individualistic social norm; conversely, when economic inequality is low it leads to an interdependent self‐construction (Sánchez‐Rodríguez et al., 2019; Sánchez‐Rodríguez, Rodríguez‐Bailón, & Willis, 2020). Similarly, the global COVID‐19 pandemic is an exceptional collective social context that might affect self‐construal. Indeed, the virus has changed our notions of “self” and whether we define ourselves as “us” (Jetten, Reicher, Haslam, & Cruwys, 2020).

In such a context, an interdependent self‐construction may be beneficial to prioritize collective obligations over personal wishes (Bavel et al., 2020). Based on previous literature we propose that the economic threat may increase interdependent self‐construal, which might lead to less tolerance towards inequality and more collective actions against it. However, interdependent self‐construal is a multidimensional construct and each dimension might be affected by different antecedents (Vignoles et al., 2016). Given that the collective economic threat linked to COVID‐19 might have generated the perception of a shared common fate (Drury et al., 2016; Drury & Reicher, 2000; Reicher, 1996), we expected that it might particularly affect feelings of self‐reliance (vs. being dependent on others), self‐containment (vs. connections with others), differentiation from others (vs. similarity), and self‐interest (vs. commitment to others). However, we have less reasons to expect that the economic threat derived by pandemic is linked with dimensions that have less to do with a perceived common fate, such as self‐direction (vs. receptiveness to influence), self‐expression (vs. harmony), and consistency (vs. variability) of self across time. Therefore, we focus only on those dimensions of self‐construal that we consider might have been affected by perceiving that the COVID19 pandemic represents a shared grievance.

## 4 THE PRESENT RESEARCH

We situate our research in the context of Spain during the first two waves of the Covid‐19 pandemic (May–October 2020). A total of 239.429 people diagnosed with covid were reported (May 31, 2020, start of the first wave), being the third country in Europe with more confirmed cases after Russia and the United Kingdom (Ministry of Health, Government of Spain, 2020a). In the second wave after summer, a total of 1.098.320 infected were reported (26 October), being, in the same way, the third country with the most confirmed cases after Russia and France (Ministry of Health, Government of Spain, 2020b). In terms of economic consequences, the pandemic could bring Spain back to inequality levels similar to those experienced during the economic crisis in 2008 (Oxfam, 2021). This translated into an increase in the relative poverty in Spain of 22.9% (1 million more people below the poverty line). The unemployment caused by the pandemic is the main generator of inequality and poverty, due to the fall in the income of the most precarious workers. The increase in unemployment was accentuated in migrants, young people, women, also doubling in the lowest educational levels (Oxfam, 2021).

The protests derived from this situation relate to the working‐class as an aggrieved group (e.g., Marches for dignity; Sabucedo et al., 2017). The pandemic increased the importance of social positions, situating social class and economic inequality in the centre of the public debate. Working‐class identity is a traditional identity among the politicized lower classes in Spain (especially for members of traditional left‐wing political parties). On the other hand, the emergence of the indignados movement in 2011 was supposed a political renovation in many senses (e.g., rejection of classic concepts, the emergence of new political parties…) in that context the identity of the 99% became popular. These are politicized and emerging identities in contexts where a great generalized injustice is perceived. The current research offers an opportunity to test in parallel old and new identities against economic inequality.

**FIGURE 1:**
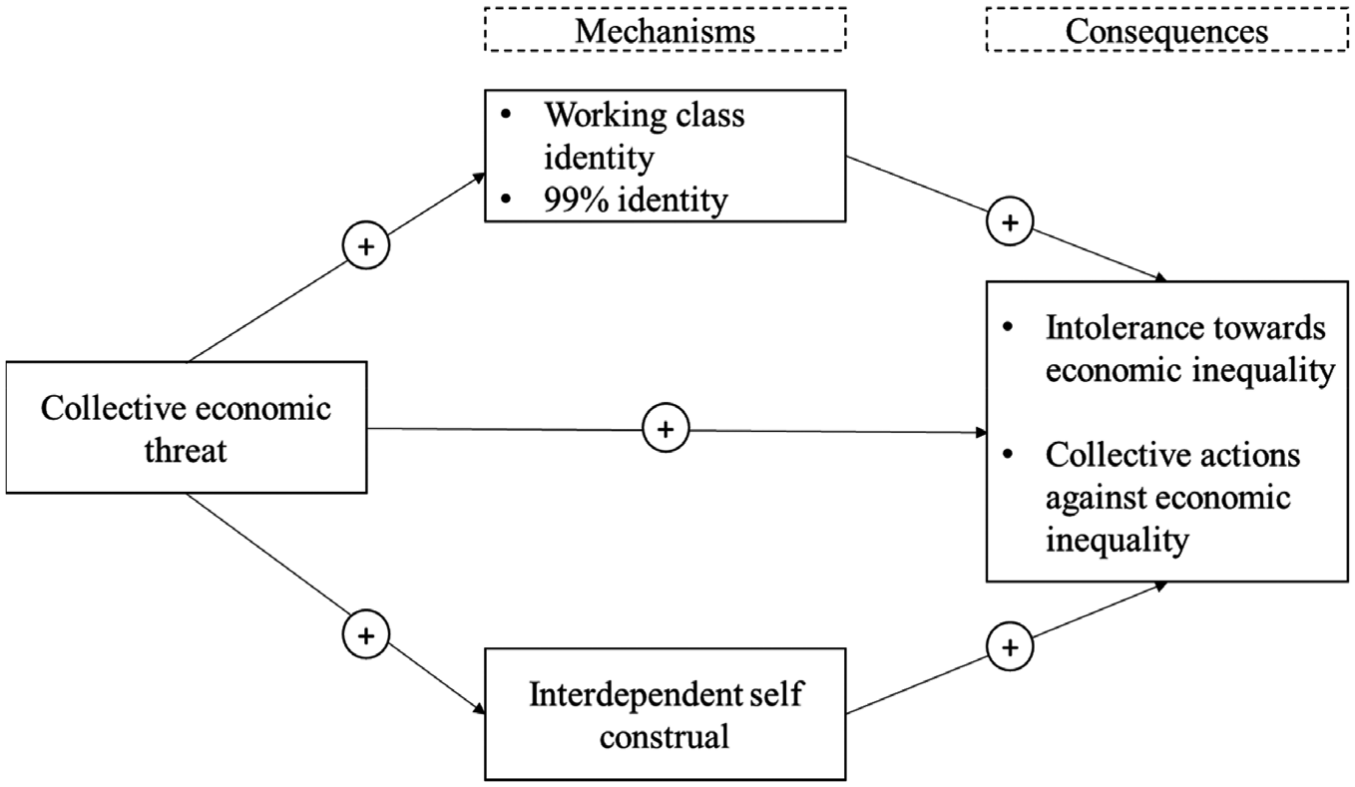
Theoretical model that shows the relationship between the collective economic threat and intolerance towards economic inequality and collective actions through identity and interdependent self‐construal mechanisms

We conducted three correlational studies to find out what kind of mechanisms people use to cope with economic threats during the COVID‐19 pandemic. We analyse the impact on class‐related politicized identity at two levels: a classical well‐established identity (i.e., the working‐class) and a new broader emerging identity (e.g., the 99%). Additionally, we investigate the role of an interdependent self‐construal as a separate path to confront this collective threat. We predicted that these two paths, identity‐based and self‐construal, are related to intolerance towards economic inequality and collective actions (Figure 1).

## 5 STUDY 1

In Study 1 (https://osf.io/sr2nz/?view_only=95056997b7d7411d8be6be0b1dd16ab2), we explored whether the economic threat generated by the Covid‐19 pandemic is related to less tolerance for inequality and support for social change (Spain, May 2020). Specifically, we tested whether the perception of economic threat is related to emerging identities against inequality—that is, the 99% identity, working‐class identity— and an interdependent self‐construal. We also tested whether this, in turn, is related to greater intolerance towards economic inequality and participation in collective actions against inequality.

## 6 METHOD

### 6.1 Participants and procedure

An incidental sampling was carried out with a general population in Spain. The study was conducted through a web link on the *Qualtrics* platform, and the collaboration was voluntary and anonymous. Fifty euros were raffled to encourage participation.

We planned to collect a minimum of 300 and a maximum of 400 observations after exclusions. After removing incomplete data, 368 participants took part in the study. We excluded five participants from the data analyses because they did not have the preregistered exclusion criteria requirements: data from participants who are not Spanish speakers. The final sample consisted of 363 participants (262 women, 96 men and 5 “other”) aged between 18 and 70 years (*M* = 33.71; *SD* = 13.96).

### 6.2 Measures^1^

**Economic threat perception**. We translated the Financial Threat Scale (FTS) (Marjanovic, Greenglass, Fiksenbaum, & Bell, 2013) and adapted it to the context of the economic threat caused by the coronavirus pandemic. The scale is made up of five items with a 5‐point scale (1 = “nothing”; 5 = “a lot/totally”).

We measured the individual threat with five items (e.g., “How much uncertainty do you feel about your economic situation?”, α = 0.91), and collective threat with three items (e.g., “How worried are you about the economic situation in Spain?*”*, α = 0.84).

**99% and working‐class identification**. First, participants read the definition of these two identities. In the case of the 99% identity, we defined it as: “The term 99% claims the majority of the world's population (99%) compared to a very small percentage (1%) that owns half of the planet's wealth (if wealth were a pie cut in two, the richest 1% takes possession of one half while the other corresponds to 99% of the world's inhabitants)”. In the case of working‐class identity, we defined it as: “The term working‐class designates the set of workers who work in exchange for a salary in opposition to the ruling class that owns the majority of the property of economic resources.”.

Afterward, we measured to what extent participants identified with the 99% (α = 0.94) and the working‐class (α = 0.88) using 3 centrality (e.g., “Being part of the 99%/working‐class is an important part of my identity”) and 3 solidarity (e.g., “I feel a bond with the 99%/working‐class”) items of Leach et al.'s identification scale (2008), and a general item (e.g., “I identify with the 99%/working‐class”) on a Likert scale (1 = “Not at all”; 7 = “Very much”).

**Interdependent self‐construal**. We measured four components of Self Construal Scale: self‐sufficient versus dependent on others (e.g., “I would rather be self‐sufficient than depend on others”), autonomy versus connections with others, (e.g., “I consider that my happiness is independent of the happiness of my friends and family”), different versus similar (e.g., “I am a unique individual”), self‐interest versus commitment to others items (e.g., “I would sacrifice my own interest for the benefit of my group”) (α = 0.71). We used four items of each component with the highest factor weight (Vignoles et al., 2016) using a 5‐point scale (1 = “It does not describe me at all”; 5 = “It describes me exactly”).

**(In)tolerance towards economic inequality**. We used a Spanish version of the Support for Economic Inequality Scale (Wiwad et al., 2019, for example, “Economic inequality is causing many of the problems in Spain”, α = 0.76) using a Likert scale (1 = “Totally disagree”; 7 = “Totally agree”).

**Collective actions**. Based on previous measures used in the literature to evaluate collective actions, formal political participation and activism (Becker, Wright, Lubensky, & Zhou, 2013; Ekman & Amnå, 2012), we created 11 items to measure willingness to participate in collective actions within the framework of economic inequality caused by the pandemic (e.g., “I would participate in peaceful protests that demand the expropriation and nationalization of all private health companies to improve health care for the entire population”; α = 0.91) using a Likert scale (1 = “Never”; 7 = “Very often”).

**Sociodemographic measures**. Finally, some sociodemographic data were requested: Subjective socioeconomic status (Adler, Epel, Castellazzo, & Ickovics, 2000), political ideology measured with one item (1 = “Extreme left”; 100 = “Extreme right”), objective social class based on the level on education and income (e.g., “How much net monthly income do you have? Consider sources of income including wages”), sex, age, mother tongue and nationality. Sex, age, political orientation and subjective economic status are used as covariates in our analyses.

In addition, we measured other variables (e.g., health threat perception, humanity identification, justification of the economic system and orientation to social dominance) not included in the text, but described in Data S1.

## 7 RESULTS AND DISCUSSION

Pearson's correlations between the main variables and descriptive statistics are presented in Table 1.

**TABLE 1:** Descriptive statistics (means and standard deviations) and bivariate correlations between the variables measured in Study 1

	Collective ET	Individual ET	Working class id.	99% id.	Inter. S‐C	Intolerance EI	Collective actions
Collective ET	4.16(0.81)	0.21**	0.31**	0.17**	0.16**	0.21**	0.16**
Individual ET		3.03(1.07)	0.01	0.01	−0.07	0.04	0.02
Working class id.			5.97(1.17)	0.25**	0.09	0.32**	0.31**
99% id.				5.09(1.79)	0.01	0.23**	0.26**
Inter. S‐C					3.21(0.47)	0.14**	0.18**
Intolerance EI						5.90(1.00)	0.53**
Collective actions							4.88(1.40)

*Note*: Diagonal shows mean of the participants' score on the scale and standard deviation in brackets.

Abbreviations: ET, Economic Threat; Id, Identification; EI, Economic Inequality; Inter. S‐C, Interdependent Self‐Construal.

** *p* ≤ .01.

Individual economic threat did not predict significantly intolerance towards economic inequality *β* = −.03, *p* = .542, neither collective action *β* = .06, *p* = .185 (see Data S1). Therefore, we only focus on collective economic threats in the subsequent studies.

We carried out two parallel mediation analyses with PROCESS (model 4; Hayes & Scharkow, 2013) to test the role of the 99% identity (M1), working‐class identity (M2) and interdependent self‐construal (M3) as potential mediators of the relationships between collective economic threat (X) and intolerance towards economic inequality (Y1) and collective actions (Y2). We used 5,000 bootstrap samples to estimate bias‐corrected standard errors and 95% percentile confidence intervals for the indirect effects. Specifically, we included the covariates sex, age, political orientation and subjective economic status in our main analyses^2^.

Concerning the first model, we found that collective economic threat was related to intolerance towards economic inequality directly and indirectly via working‐class and 99% identification, but not via interdependent self‐construal. Secondly, we found that collective economic threat was related to collective actions directly and indirectly via 99% identification. On the contrary, it was not mediated by working‐class identification, or by interdependent self‐construal (see total, direct and indirect effects in Table 3).

These results provided initial evidence that collective economic threat is related to attitudes towards economic inequality and willingness to participate in collective actions through the activation of politicized identities. Nevertheless, we should note that the confidence intervals of our indirect effects were close to zero, so we decided to confirm the social identity path and again explore the interdependent self‐construal path in further studies.

## 8 STUDY 2

In Study 2 (https://osf.io/auh4r/?view_only=9b9645316e1c4167bcd09d32447ec03a) we preregistered a confirmatory study in the second wave of the COVID‐19 pandemic (Spain, October 2020). We expect that the relation between collective economic threat and intolerance towards economic inequality is mediated by identification with the working class (Hypothesis 1a), and by identification with 99% (Hypothesis 1b). Similarly, we expect that the relation between collective economic threat and collective actions is mediated by identification with the 99% (Hypothesis 2). Further, with exploratory purposes, we analyse the working‐class identity and interdependent self‐construal as mediators in our models^3^.

Hypothesis 1a *The relation between collective economic threat and intolerance towards economic inequality is mediated by identification with the working class*.

Hypothesis 1b *The relation between collective economic threat and intolerance towards economic inequality is mediated by identification with the 99%*.

Hypothesis 2 *The relation between collective economic threat and collective actions is mediated by identification with the 99%*.

## 9 METHOD

### 9.1 Participants and procedure

The procedure was similar to Study 1. To achieve a power of 0.80 (considering an alpha level of 0.05), for detecting a medium‐large effect size on path a = 0.40 and for detecting a medium effect size on path b = 0.20, we needed at least 202 participants (Fritz & MacKinnon, 2007). We planned to recruit a minimum of 250 participants to further increase our statistical power.

Seven hundred and two participants finished the study, of which we excluded 36 participants from the data analyses because they did not have the preregistered exclusion criteria requirements: data from participants who are not Spanish speakers. The final sample consisted of 666 participants. As we planned to recruit 250 participants for Study 2, we decide to use the first 250 participants in date order to corroborate the hypotheses referred to in Study 2 and analyse the rest as confirmatory in Study 3.

### 9.2 Measures

Using the same measures described in Study 1 we evaluated collective economic threat perception (α = 0.79), individual economic threat (α = 0.90), 99% identification (α = 0.95), working‐class identification (α = 0.88), interdependent self‐construal (α = 0.70), intolerance towards economic inequality (α = 0.76), collective actions (α = 0.91) and sociodemographics. In addition, we measured other variables that were excluded from the main text (e.g., emotions; group efficacy; community‐focused collective actions; see Data S1).

## 10 RESULTS AND DISCUSSION

We carried out Pearson's correlations between the main variables and descriptive statistics (see Table 2).

**TABLE 2:** Descriptive statistics and bivariate correlations between the variables measured in Study 2 and 3

	Study 2 M (SD)	Study 3 M (SD)	Collective ET	Individual ET	Working class id.	99% id.	Inter S‐C	Intolerance EI	Collective actions
Collective ET	4.05(0.77)	4.03(0.81)	—	0.22**	0.16**	0.12*	0.03	0.26**	0.10*
Individual ET	3.45(0.94)	3.42(0.98)	0.26**	—	0.09	0.03	−0.07	0.20**	0.12*
Working class id.	5.65(1.28)	5.52(1.38)	0.15*	0.09	—	0.03	0.04	0.34**	0.33**
99% id.	4.80(1.91)	4.27(2.00)	0.09	0.10	0.05	—	0.01	0.07	0.19**
Inter S‐C	3.03(0.45)	3.04(0.43)	0.14*	0.16*	0.11	0.14*	—	0.07	0.12**
Intolerance EC	6.01(1.00)	5.85(1.07	0.16*	0.07	0.35**	0.23**	0.20**	—	0.51**
Collective actions	4.54(1.45)	4.54(1.45)	0.01	0.18**	0.39**	0.08	0.17**	0.46**	—

*Note*: The results of Study 2 are below the diagonal and the results of Study 3 are above the diagonal.

Abbreviations: ET, Economic Threat; Id, Identification; EI, Economic Inequality; Inter S‐C, Interdependent Self‐Construal.

* *p* ≤ .05;

** *p* ≤ .01.

Similar to Study 1, we conducted two parallel mediation analyses^4^ with PROCESS (model 4; Hayes & Scharkow, 2013) to test our pre‐registered hypotheses.

Results showed that collective economic threat was related to intolerance towards economic inequality directly and indirectly with identification with the workingclass (supporting Hypothesis 1a) but not with identification with the 99% (against Hypothesis 1b) or interdependent self‐construal. In addition, collective economic threat was not directly related to collective actions but indirectly to workingclass identification. On the contrary, it was not mediated by identification with the 99%, thus providing no support for Hypothesis 2, or by interdependent self‐construal (see total, direct and indirect effects in Table 3).

**TABLE 3:** Summary of total, direct and indirect effect of collective economic threat and intolerance towards economic inequality, collective actions, mediated by 99% and working‐class identification, Studies 1–2–3 and pooled analyses

	Study 1 (*N* = 363)				Study 2 (*N* = 250)				Study 3 (*N* = 416)				Pooled analyses (*N* = 1,029)			
	Effect	SE	LLCI	ULCI	Effect	SE	LLCI	ULCI	Effect	SE	LLCI	ULCI	Effect	SE	LLCI	ULCI
Total effect	0.23	0.06	0.12	0.35	0.23	0.07	0.09	0.37	0.32	0.06	0.21	0.43	0.21	0.03	0.16	0.27
Direct effect	0.12	0.06	<0.01	0.24	0.15	0.07	0.02	0.30	0.29	0.06	0.18	0.41	0.16	0.03	0.11	0.22
IE working class	0.07	0.03	0.02	0.14	0.03	0.02	<0.01	0.07	0.03	0.02	0.01	0.07	0.03	0.01	0.02	0.05
IE 99%	0.02	0.01	<0.01	0.05	0.02	0.01	<−0.01	0.05	−0.01	0.01	−0.02	0.01	0.01	<0.01	<0.01	0.02
IE interdependent self‐construal	0.02	0.01	<−0.01	0.05	0.03	0.02	<−0.01	0.06	<−0.00	0.01	−0.01	0.01	0.01	<0.01	<0.01	0.02
Total effect	0.26	0.08	0.11	0.41	0.04	0.10	−0.16	0.24	0.11	0.07	−0.04	0.25	0.09	0.03	0.03	0.14
Direct effect	0.16	0.08	0.01	0.32	−0.04	0.10	−0.24	0.15	0.05	0.07	−0.10	0.19	0.04	0.03	−0.01	0.09
IE. Working class	0.04	0.03	−0.02	0.12	0.04	0.03	<0.01	0.11	0.04	0.02	0.01	0.09	0.03	0.01	0.01	0.04
IE. 99%	0.03	0.02	<0.01	0.07	−0.01	0.01	−0.04	0.02	0.02	0.01	<0.01	0.05	0.01	<0.01	<0.01	0.02
IE. Interdependent self‐construal	0.02	0.02	<−0.01	0.06	0.05	0.02	0.01	0.10	<−0.01	0.01	−0.02	0.02	0.01	<0.01	<0.01	0.02

Abbreviation: *IE* = indirect effect.

The results of Study 2 showed that collective economic threat was related to identification with the working class and this in turn with a greater intolerance and participation in collective actions against economic inequality. Furthermore, we find this relationship to be mediated by the interdependent self‐construal, but not by the 99% as we had predicted based on Study 1's results.

## 11 STUDY 3

In Study 3 (https://osf.io/mqbd3/?view_only=f4c27c8c8db14ba8ab2375fa35b1a40a) (Spain, October 2020) we wanted to confirm the role of working‐class identity as a mediator of the impact of collective economic threat on intolerance towards economic inequality (Hypothesis 1a) and confirm its role in the support of collective action after the economic threat (Hypothesis 2a). Also, the role of interdependent self‐construal, as a second parallel mediator in explaining both intolerances towards economic inequality (Hypothesis 1b) and collective action against economic inequality (Hypothesis 2b) as a context of economic collective threat. We explored the role of 99% identification as a mediator to clarify the inconsistencies found in Studies 1 and 2.

## 12 METHOD

### 12.1 Participants and procedure

The procedure was the same as in Study 2. The final sample of Study 3 consisted of 416 participants (286 women, 123 men, 7 other) aged between 18 and 62 (*M* = 22.88, *SD* = 5.66).

### 12.2 Measures

Using the same measures described in Study 2 we evaluated collective economic threat perception (α = 0.85), individual economic threat (α = 0.88), 99% identification (α = 0.90), working‐class identification (α = 0.90), interdependent self‐construal (α = 0.68), (in)tolerance towards economic inequality (α = 0.74), collective actions. (α = 0.91) and sociodemographics.

## 13 RESULTS AND DISCUSSION

We carried out Pearson's correlations between the main variables and descriptive statistics (see Table 2).

Similar to Study 2 we conducted two parallel mediation analyses with PROCESS (model 4; Hayes & Scharkow, 2013).

The results showed that collective economic threat was related to intolerance towards economic inequality directly and indirectly via working‐class identification, which supports Hypothesis 1a. However, it was not mediated via interdependent self‐construal, which is contrary to Hypothesis 1b, or by 99% identification. In addition, the collective economic threat was not related directly to collective actions but indirectly via working‐class identification (supporting Hypothesis 2a) and 99% identification. On the contrary, it was not mediated by interdependent self‐construal, thus providing no support for Hypothesis 2b (see total, direct and indirect effects in Table 3).

Across studies, there was evidence that working‐class identity mediated the relation between the collective economic threat and our criterion variables: intolerance towards economic inequality and collective actions. However, the results regarding 99% identification and interdependent self‐construal as mediators are less consistent. Given the heterogeneity of some results, we decided to conduct a pooled analysis of the three studies to confirm the patterns that hold across samples.

### 13.1 Pooled analysis

To provide insights into the robustness of the central effects we pooled the data of Studies 1–3 following an integrative data analysis approach (Curran & Hussong, 2009). The total sample was composed of 1,029 participants, (721 women, 291 men, 17 other) aged between 18 and 70 (*M* = 26.79, *SD* = 10.75). We carried out the same analysis strategy as in Studies 1–3 and tested prerregistered Hypotheses 1 and 2 of Studies 2–3.

We conducted a sensitivity analysis for a mediation analysis with three mediators using “pwr2ppl” package for RStudio (Aberson, 2019) to determine the detectable effect size. With α = 0.05 and power 1‐β (M1 = 0.80; M2 = .80; M3 = 0.80), and with 1,029 participants, we are able to detect a minimum effect size between *r* = 0.10 and *r* = 0.15. As such, we think we have enough power to detect the hypothesized effects.

Results showed that collective economic threat was related to intolerance towards economic inequality directly and indirectly via working class and 99% identification and interdependent self‐construal, supporting the model proposed in Hypothesis 1. In the same way, collective economic threat was related to collective actions indirectly (but not directly) via working class and 99% identification and interdependent self‐construal, supporting the model proposed in Hypothesis 2.

**FIGURE 2:**
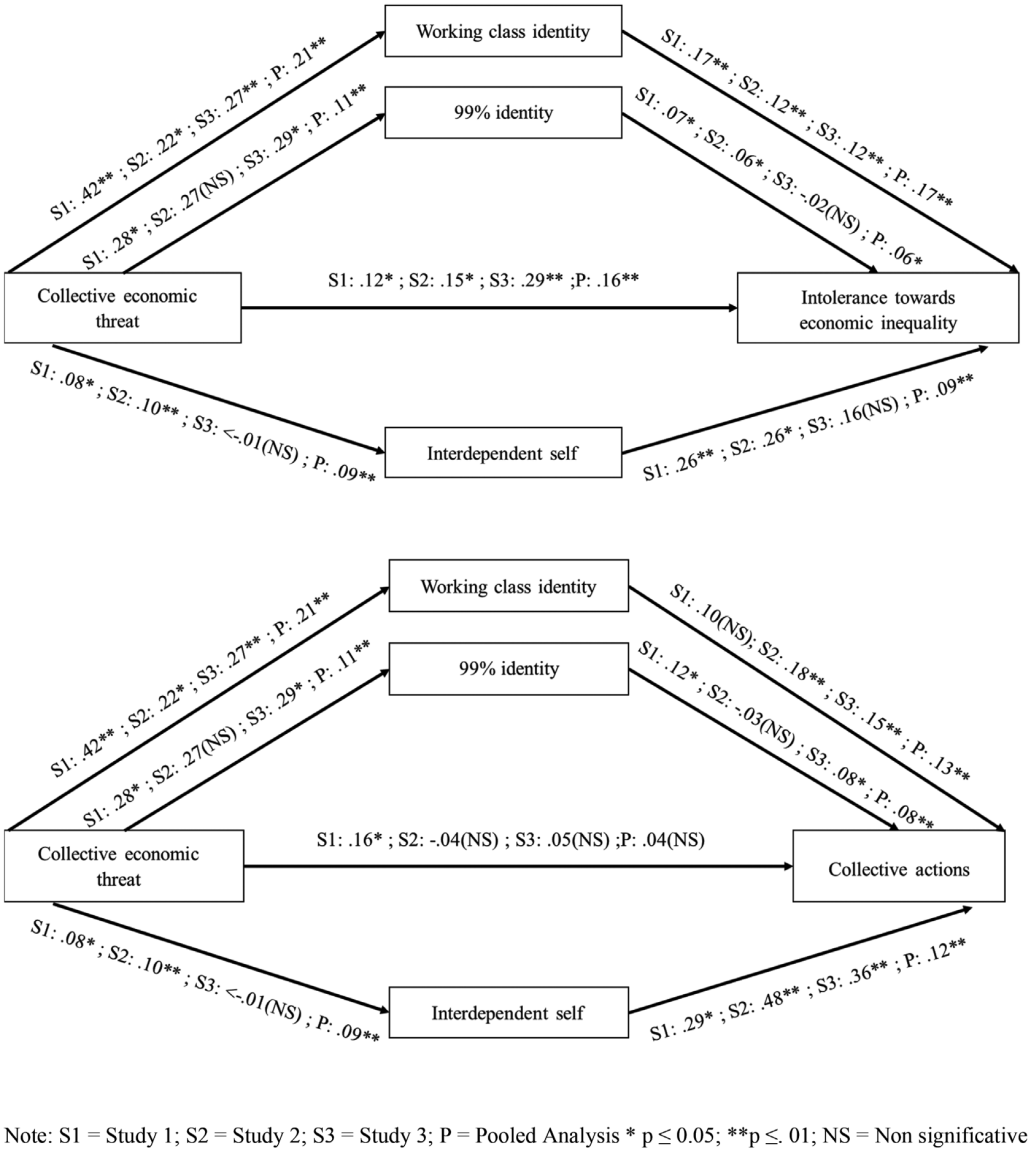
Identification with the working class, 99% identities and interdependent self‐construal as mediators between collective economic threat, intolerance towards economic inequality and collective actions in Studies 1–3 and pooled analysis. S1 = Study 1; S2 = Study 2; S3 = Study 3; P = Pooled Analysis *p ≤ .05; **p ≤ 0.01; NS = Non significative.

A summary of the results appears in Table 3 and Figure 2.

In sum, the results of the pooled analyses confirmed that the social identity and interdependent self‐construal paths contribute to understanding the relationship between collective economic threat in the context of the COVID‐19 pandemic and responses against inequality such as intolerance towards economic inequality and collective actions (Figure 2).

## 14 GENERAL DISCUSSION

Among the possible ways to deal with economic threat, our results show evidence of two possible mechanisms to cope with it: via social identification and interdependent self‐construal processes. Specifically, we analysed how these two mechanisms are fuelled by collective economic threat and, in turn, are related to a greater intolerance towards economic inequality and collective actions against it. Despite the negative consequences of the pandemic, based on our results we argue that this pandemic has reinforced the sense of community and, above all, has promoted a rejection of inequality.

In this paper we made two important contributions. First, we showed that politicized identities linked to economic inequality serve to channel collective efforts to deal with economic threat. COVID‐19 threat may induce or exacerbate intergroup tensions and hostility (Bavel et al., 2020) but the perception of a shared or common identity with various social groups during the pandemic can prevent such tensions (Dovidio, Ikizer, Kunst, & Levy, 2020). The economic threat derived from the pandemic implies a shared fate, but the different effects of such threats on people—depending on their social and economic status—might be perceived as a shared injustice, however, future research should address this directly. When there is an injustice or shared grievance, this identity also has a politicized value, further increasing the value of cohesion that it mobilizes for protests (van Zomeren et al., 2008). This is especially relevant if we focus on social class as a representative element of people's identities (Easterbrook et al., 2020; Manstead, 2018). The working class has been marginalized, eroding its communal and collective aspects (Jones, 2011). This reduces the salience and clarity of traditional classes (Leach et al., 2008), promoting the perception that status difference is an individual—instead of a collective—process (Jetten, Iyer, Branscombe, & Zhang, 2013). Our results suggest that class identities that promote social change arise in response to the pandemic economic threats.

Second, we found evidence that social change is also fuelled by changes at the self‐construal level (Markus & Kitayama, 1991; Singelis, 1994). People who perceived a high collective threat also showed a higher interdependent self‐construal, in line with previous research in other contexts (Oishi & Komiya, 2017). Interdependent self‐construal, in turn, was related to an increase in intolerance towards inequality and participation in collective actions. These results suggest that seeing yourself as connected to others, fitting in with others, perceiving yourself as similar, and sacrificing personal goals may trigger shared goals to face inequalities. We should note that we did not include all the dimensions of interdependent self‐construal, just those that we expected were related to the idea of common fate.

It is also important to highlight that in these studies we use social identity and self‐construal as two different and independent processes. People with an interdependent self‐construal may have a greater tendency for thinking in group—rather than in individual— terms (Markus & Kitayama, 1991), which might suggest that social identity and interdependent self‐construal are positively related. However, under specific circumstances, those who are more independent might be the ones who identify more with social groups (McAuliffe, Jetten, Hornsey, & Hogg, 2003). Future research is needed to clarify the relations between these two constructs. Importantly, we argue that beyond the potential link between them, social identity and self‐construal can separately impact intolerance towards inequality and participation in collective actions as a response to collective economic threat.

## 15 APPLIED IMPLICATIONS

Our results suggest that people perceive a high economic threat caused by the coronavirus pandemic, particularly the collective economic threat. Thus, individuals are not only worried about health issues caused by the pandemic, but also about the economy. However, the media focus their messages about the pandemic on health‐related issues undermining the socio‐economic impact of it. A broader coverage of the pandemics implications directly addressing social inequalities would help to promote active coping.

Moreover, economic threat triggers collective actions via class identity and an interdependent self. Thus, social awareness of economic threats can be a tool for social movements to mobilize people to protest against economic inequality and build a sense of community. This is a socially constructive response that contravenes the tendency to increase prejudice and ethnocentrism as a consequence of the Covid threat (Lemay et al., 2021). Therefore, we emphasize the benefits of promoting this route to social coping via awareness of shared economic grievances and social class identities instead of focusing on other levels of categorization (e.g., nationality).

## 16 LIMITATIONS

Among the possible limitations of our study, the indirect effects of shared identity, especially the 99% identity, and interdependent self‐construal as mediators are small and inconsistent across studies. Even so, this is solved in a parsimonious way in the pooled analysis. Importantly, all our results are correlational, so the inference about causality is limited. Furthermore, in these contexts of polarization some disruptive individual identities for social change become relevant (e.g., covid‐deniers) that should be tested in further research. Finally, a more diverse sample in other contexts could help to corroborate the generalizability of our findings. However, the data collection occurred at an extraordinary time and it is a challenge to know if these results could be replicated in another context.

In summary, the economic threat derived from COVID‐19 pandemic (in its first stages) is related to an increase in prosocial and conflict responses—a greater intolerance towards economic inequality and a greater involvement in collective actions to reduce inequalities. In this process, the activation of politicized identities (e.g., classic identities, working class; and the new 99%) and interdependent self‐construal play a key role. This can lead to a stronger bond with others and a greater awareness of the needs of others, which can allow us to jointly face multiple challenges in our societies.

## FUNDING INFORMATION

This research has been funded by MCIN/AEI/10.13039/501100011033 (Grant no.PID2019‐111549GB‐I00) and by FEDER/Junta de Andalucía‐Consejería de Transformación Económica, Industria, Conocimiento y Universidades (Grant no. A‐SEJ‐72‐UGR20). Funding for open access charge: Universidad de Granada / CBUA.

## CONFLICTS OF INTEREST

The authors declare that there are no potential conflicts of interest with respect to the research, authorship, and/or publication of this article.

## ETHICS STATEMENT

The reported studies were approved by the ethical committee of the University of Granada (Ethics Clearance ID: 1410/CEIH/2020).

## INFORMED CONSENT

All participants signed anonymous consent to participate in both studies. All authors consent to the publication of this paper.

## Supporting information


**Data S1** Supporting information.

**Data S2** Supporting information.

## Data Availability

The data that support the findings of this study are openly available in OSF at https://osf.io/e3hk6/?view_only=92ecabf44f4c47e6b727180bc418b45f.

## Endnotes

^1^
All pre‐registered measures are translated into English in the Data S1.

^2^
We introduced political orientation to control for the existing overlap between politicized identification and political ideology (de Moreira et al., 2018). Further, we added subjective economic status (SES) as a covariate to control for the structural aspect of class in the participants. SES, age and sex are important predictors of the importance that people place on different types of identities within their self‐concepts (Easterbrook et al., 2020), and are related to our criterion variables (García‐Sánchez, Osborne, Willis, & Rodríguez‐Bailón, 2020) so we included them to adjust for potential background influences.

^3^
We did not prerregister that collective economic threat will increase collective action intentions through the activation of the working class identification due to the results of Study 1. However, we conducted this exploratory analysis because it is theoretically relevant. In addition, we wanted to explore the interdependent self‐construal as a mediator of collective actions focused on the community.

^4^
We have decided to include the models in parallel instead of simple ones to simplify the presentation. The results in parallel models were similar to simple models.
