# Supplementary material for: Fighting inequalities in times of pandemic: The role of politicized identities and interdependent self‐construal in coping with economic threat
Source: J Community Appl Soc Psychol. 2022 Jun 13:10.1002/casp.2632. Online ahead of print. doi: 10.1002/casp.2632 (PMC9349427; doi:10.1002/casp.2632)
Supplement: Supplementary file 1 — Data S1 Supporting information. [file CASP-9999-0-s002.docx]

**Supplementary Materials**

**pertaining to**

**Fighting inequalities in times of pandemic: The role of the politicized identities and interdependent self-construal in coping with economic threat**

This section contains the supplementary material in addition to the manuscript. (1) Specifically, measures, hypotheses and analyses of Studies 1-3 that have been discarded to facilitate understanding from the original manuscript for being inconsistent.

Study 1: Measures (e.g., health threat perception; humanity identification; orientation to social dominance; justification to economic system) and descriptive statistics and correlation.

Study 2-3: Measures (e.g., collective efficacy; emotions; Community collective actions), hypothesis discarded and parallel mediation analyses.

(2) You can also find the **Social and** **Community Impact Statement**: (a) a Relevance Statement of up to 150 words that explains why the article is relevant to communities; (b) practical indications and examples on how to implement the content of the article in community settings. In English and Spanish

(3) In the final section of this document all the measurement materials appear, including the items used for each variable.

1. DESCRIPTION OF ADDITIONAL MEASURES AND RESULTS

**Study 1**

**Measures**

Here we discuss measures that were included in the first study that are not described in the main text

**Health threat perception.** We translated and adapted the Financial Threat Scale (FTS) (Marjanovic et al., 2013) to the context of the health threat caused by the coronavirus pandemic. The scale is made up of five items with a Likert scale from 1 to 5. We measured health threat perception. On the one hand, we measured the personal threat with five items (e.g., “How much uncertainty do you feel about your health?*”,* α = .88), and on the other hand, the collective threat with three items (e.g., “How worried are you about the health situation in Spain?”, α = .84).

**Humanity identification.** First, we defined humanity identity “The term humanity comes from a Latin word related to the nature of the human race. It serves to mention the set of human beings that inhabit the planet”. After, we measured, with a Likert scale (1 = Not at all; 7 = Very much) to what extent did participants identify with humanity (α = .89). We measured humanity identification with the centrality and solidarity items of Leach et al., (2008) scale adapted to humanity identity. Seven items made up the measured; three captured the idea of centrality (e.g., “The fact of being part of humanity is an important part of my identity”), and three more captured the idea of solidarity (e.g., “I feel a bond with humanity”). In addition, we included a general item (e.g., “I identify with (in-group)”).

**Orientation to social dominance.** We used the Social Dominance Orientation scale (SDO) (Pratto et al., 1994) translated into Spanish. The scale consisted of sixteen items (Alpha = .82) formed by two main components; Group dominance (α = .72) (e.g., “*The value of some groups of people is greater than that of others*”) and Opposition to equality (Alpha = .84) (e.g., “We should do our best to equalize the conditions for different groups”), with a Likert scale (1 = Totally disagree; 7 = Totally agree).

**Justification of the economic system.** To measure the general ideological tendency to legitimize economic inequality we used a reduced version of the original Economic System Justification scale (ESJ, Jost & Thompson, 2000) adapted and validated into Spanish (Jaume et al., 2012). The scale consisted of seven items (α = .79) (e.g., “If people work hard, they almost always get what they want”) with a Likert scale (1 = Totally disagree; 7 = Totally agree).

**Results**

A summary of the descriptive statistics and correlation between the variables of our Study 1 is presented in Table 1.

**Table 1.** Descriptive statistics and bivariate correlations between the variables measured in Study 1.

|  | CET | IET | CHT | IHT | WC Id. | 99% Id. | H id. | Inter S-C | Intol EI | Coll actions | OSD | JES |
| --- | --- | --- | --- | --- | --- | --- | --- | --- | --- | --- | --- | --- |
| CET | 4.15(0.81) | .21** | .47** | .29** | .31** | .17** | .01 | .16** | .21** | .16** | -.07 | .01 |
| IET |  | 3.03(1.07) | .10 | .09 | .01 | .01 | -.08 | -.07 | .04 | .02 | .12* | .07 |
| CHT |  |  | 3.97(0.84) | .46** | .25** | .14* | .12* | .10* | .17** | .19** | -.07 | .01 |
| IHT |  |  |  | 2.85(0.93) | .19** | .12* | .10 | .12* | -.02 | .01 | .08 | .17** |
| WC Id. |  |  |  |  | 5.97(1.17) | .25** | .21** | .09 | .32** | .31** | -.20** | -.14* |
| 99% Id. |  |  |  |  |  | 5.09(1.79) | .16** | .01 | .23** | .26** | -.11* | -.09 |
| H id. |  |  |  |  |  |  | 6.03(1.16) | .20 | .07 | .11* | -.21** | -.03 |
| Inter S-C |  |  |  |  |  |  |  | 3.12(0.47) | .14** | .18** | -.14** | -.18** |
| Intol EC |  |  |  |  |  |  |  |  | 5.90(1.00) | .53** | -.54** | -.39** |
| Coll actions |  |  |  |  |  |  |  |  |  | 4.88(1.40) | -.45** | -.42** |
| OSD |  |  |  |  |  |  |  |  |  |  | 2.07(0.77) | .44** |
| JES |  |  |  |  |  |  |  |  |  |  |  | 2.40(0.74) |

*Note*: * *p* ≤ 0.05; ***p* ≤ 0.01; C*ET*., Collective Economic Threat; *IET*., Individual Economic Threat; *CHT*., Collective Health Threat; *IHT*., Individual Health Threat; *WC Id*., Working class identification; *99% id*., 99% identification; *H id*., Humanity identification; *Inter. S-C*., Interdependent Self-Construal; *Intol EC*., Intolerance towards Economic Inequality; *Coll actions*., Collective actions; *OSD*., Orientation to Social Dominance; *JES*., Justification of Economic System. Diagonal shows mean of the participants’ score in the scale and standard deviation in brackets.

**Study 2 & 3**

**Pre-registered hypothesis discarded:**

Hypothesis 3. We predicted two indirect effects in parallel. The relation between the collective economic threat and the community collective actions are mediated, in the one hand, by the levels of identification with the identity of the working class, and on the other hand, by the interdependent self-construal. In the sense that the perception of economic collective threat because of COVID-19 will lead participants to identity more strongly with the working class and to show more interdependent self-construal, and both will lead to increase the willingness to participate in community collective actions.

Hypothesis 4, 5 y 6. We predicted a significant mean difference on the participant’s perception of illusion, hope and indignation between the first and second waves of pandemic. We expected that the emotions of illusion and hope that the participants remember having felt in the first wave of the pandemic will be significantly greater that the emotions of illusion and hope that participants felt in the second wave of the pandemic. Also, the emotions of indignation that the participants remember having felt in the first wave of the pandemic will be significantly lower than the emotions of indignation that participants felt in the second one.

Hypothesis 7. We predicted a significant mean difference of collective efficacy between the first and second waves of pandemic. We expected that the collective efficacy that the participants remember having perceived in the first wave of the pandemic will be significantly greater than the collective efficacy that the participants perceived in the second one.

***Measures***

In the same way as in Study 1, some measures and results did not appear in the main text. Here we discuss measures that were included in the Study 2-3 that are not described in the main text

**Collective efficacy.** We used four items translated into Spanish (Stolleberg et al., 2015) (e.g., “Together, the citizens, we are stronger”). We measured in two different ways. First, we asked participants to recall the first wave of the pandemic (Study 2: α = .76; Study 3: α = .82) and, second, we asked how they perceive it in the current wave (Study 2: α = .82; Study 3: α = .84).

**Emotions.** In addition, we included three items to measure hope (“Hope that as a society a change of course will be considered”) (Study 2: r = .18 p = .005; Study 3: r = .211, p < .001), illusion (“Illusion that things could change”) (Study 2: *r* = .147, *p* = .021; Study 3: *r* = .133, *p* < .01) and indignation (“Indignation and the impact of the pandemic because the pandemic exacerbates economic inequality”) (Study 2: *r* = .645, *p* < .001; Study 3: *r* = .618, *p* < .001). Following the same logic than in the previous measure, we asked participants to recall the emotions that they experienced during the first wave of pandemic, and second one**.**

**Community collective actions**. We also measured community collective actions with four items (e.g. It would participate in neighbourhood groups that have been formed to help with purchases for the elderly and the population at risk; Study 2: α = .88; Study 3: α = .87).

**Results**

Some results do not appear in the main manuscript due to facilitate of understanding or inconsistent results.

***Individual and collective economic threat antecedents***

. In Study 1, our results showed that the collective economic threat positively predicted both intolerance toward economic inequality *F* (6, 355) = 16.68; *p* < .001, *β* = .24, *p* <.001 and collective actions *F* (6, 355) = 35.15; *p* < .001, *β* = .15, *p* < .001. On the contrary, the individual economic threat did not predict intolerance towards economic inequality *β* = -.03, *p* = .542, neither collective actions *β* = .06, *p* = .185. Further, following the same plan analysis, we introduced the working class and the 99% identification and the interdependence self-construal as criterion variables. Results showed that the collective economic threat positively predicted working class identification *F* (6, 347) = 12.37; *p* < .001, *β* = .29, *p* <.001, 99% identification *F* (6, 331) = 2.55; *p* = .020, *β* = .13, *p* = .021 and interdependence self-construal *F* (6, 355) = 10.72; *p* < .001, *β* = .13, *p* < .01. For these reasons, we carried out the parallel mediation analysis to test the role of 99% identity, working class identity and interdependent self-construal as potential mediations between collective economic threat and intolerance towards economic inequality and collective actions. Given these results in Study 1, we focus only on collective threat and we did not pre-register the individual economic threat in the subsequent studies.

***Social identity and self-construal as mediators between collective economic threat and community collective actions***

Then, we carried out parallel mediation analyses with PROCESS (model 4; Hayes, 2013) to test the role of 99% identity (M1), working class identity (M2) and the interdependent self-construal (M3) as potential mediators of the relationships between collective economic threat (X) and the community collective action (Y). We used 5,000 bootstrap samples to estimate bias-corrected standard errors and 95% percentile confidence intervals for the indirect effects. We included the covariates: sex, age, political orientation and subjective economic status.

In Study 2, neither the total effect of collective economic threat on community collective actions (*b* = 0.14, 95% CI [-0.09, 0.36]; *p* = .237), nor the direct effect (*b* = 0.06, 95% CI [-0.17 0.29]; *p* = .613) were significant. The indirect effects via the working class identity (*b* = 0.03, 95% CI [-0.01,0.10]), and 99% identity (*b* = 0.04, 95% CI [-.01,0.04]) were not significant. Otherwise, the indirect effect via interdependent self-construal was significant (*b* = 0.04, 95% CI [0.01,0.10]).

In Study 3, we found that the total effect of collective economic threat on community collective actions was significant (*b* = 0.24, 95% CI [0.08, 0.40]; *p* = .003), also the direct effect (*b* = 0.22, 95% CI [0.05, 0.38]; *p* = .009). Neither the indirect effects via the 99% identity (*b* = -0.01, 95% CI [-0.02,0.24]), nor the indirect effect via interdependent self-construal (*b* = -0.01, 95% CI [-0.02,0.02]) were significant. Otherwise, the indirect effect via working class identity was significant (*b* = 0.03, 95% CI [0.01,0.08]).

***Differences on the perception of emotions and collective efficacy between the first and second wave of pandemic***

In Study 2, we conducted a t-test to explore possible differences on the perception of emotions (illusion, hope and indignation) as well as on collective efficacy over time. The results showed significant differences between pandemic waves on illusion, *t*(244) = 12.764, *p* < .001, *d* = 1.07; hope, *t*(244) = 13.74, *p* < .001, *d* = 1.12; and indignation, *t*(244) = -7.84, *p* < .001, *d* = .43). In such a way that participants perceived in the first pandemic wave more illusion (*M* = 5.20; *SD* = 1.81) and hope (*M* = 4.93; *SD* = 1.78) but lower indignation (*M* = 5.31; *SD* = 1.72) that in the second pandemic wave (illusion: *M* = 3.28; *SD* = 1.79; hope: *M* = 2.97; *SD* = 1.71; indignation: *M* = 5.98; *SD* = 1.36). However, differences on perceived collective efficacy between the first (*M* = 5.32; *SD* = 1.30) and the second pandemic waves (*M* = 5.15; *SD* = 1.50) were not significant, *t*(244) = 1.907, *p* = .058, *d* = .121).

The results of Study 3 showed significant differences between pandemic waves on illusion, *t*(405) = 17.28, *p* < .001, *d* = 1.116; hope, *t*(405) = 18.68, *p* < .001, *d* = 1.17; indignation, *t*(404) = -8.52, *p* < .001, *d* = 0.38; and collective efficacy *t*(404) = 5.76, *p* < .001, *d* = 0.30. In such a way that participants perceived in the first pandemic wave more illusion (*M* = 5.27; *SD* = 1.67) and hope (*M* = 5.00; *SD* = 1.80) but less indignation (*M* = 5.36; *SD* = 1.64) that in the second pandemic wave (illusion: *M* = 3.44; *SD* = 1.61; hope: *M* = 2.99; *SD* = 1.64; indignation: *M* = 5.93; *SD* = 1.36). Finally, the means of perceived collective efficacy in the first wave (*M* = 5.45; *SD* = 1.25), was higher than in the second wave (*M* = 5.04; *SD* = 1.52), *t*(404) = 5.761, *p* < .001, *d* = 0.295).

1. SOCIAL AND COMMUNITY IMPACT STATEMENT

English language:


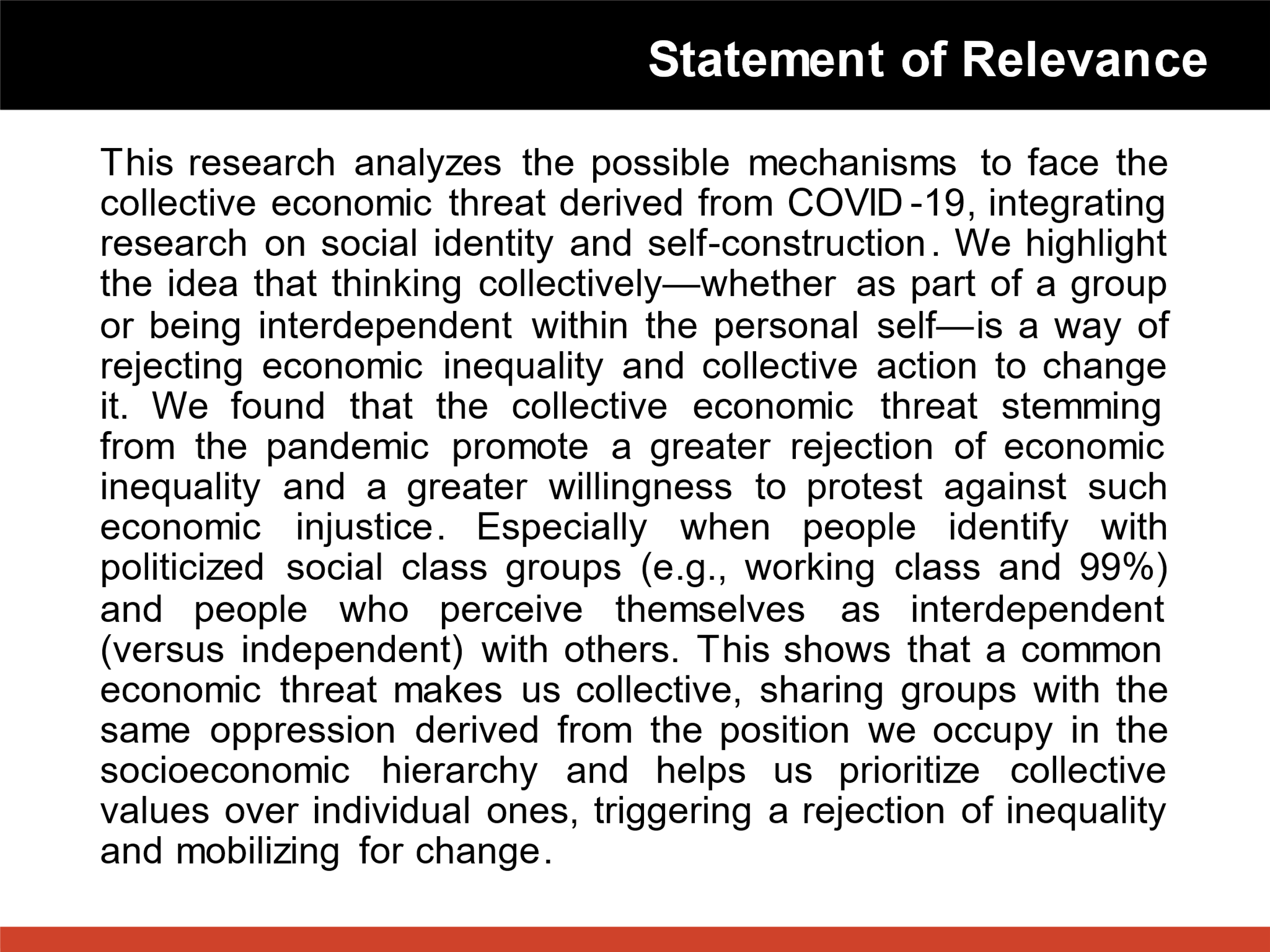


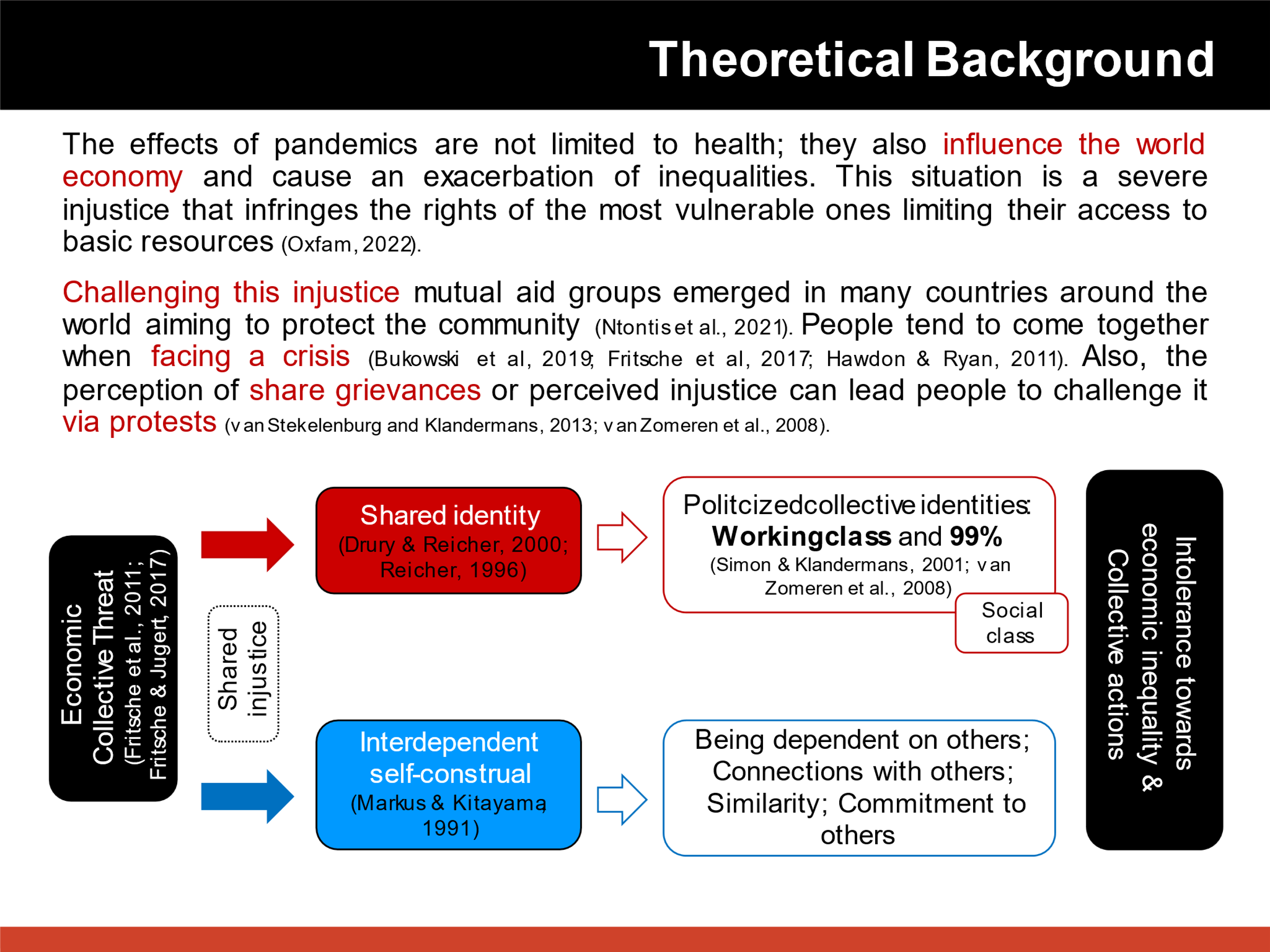


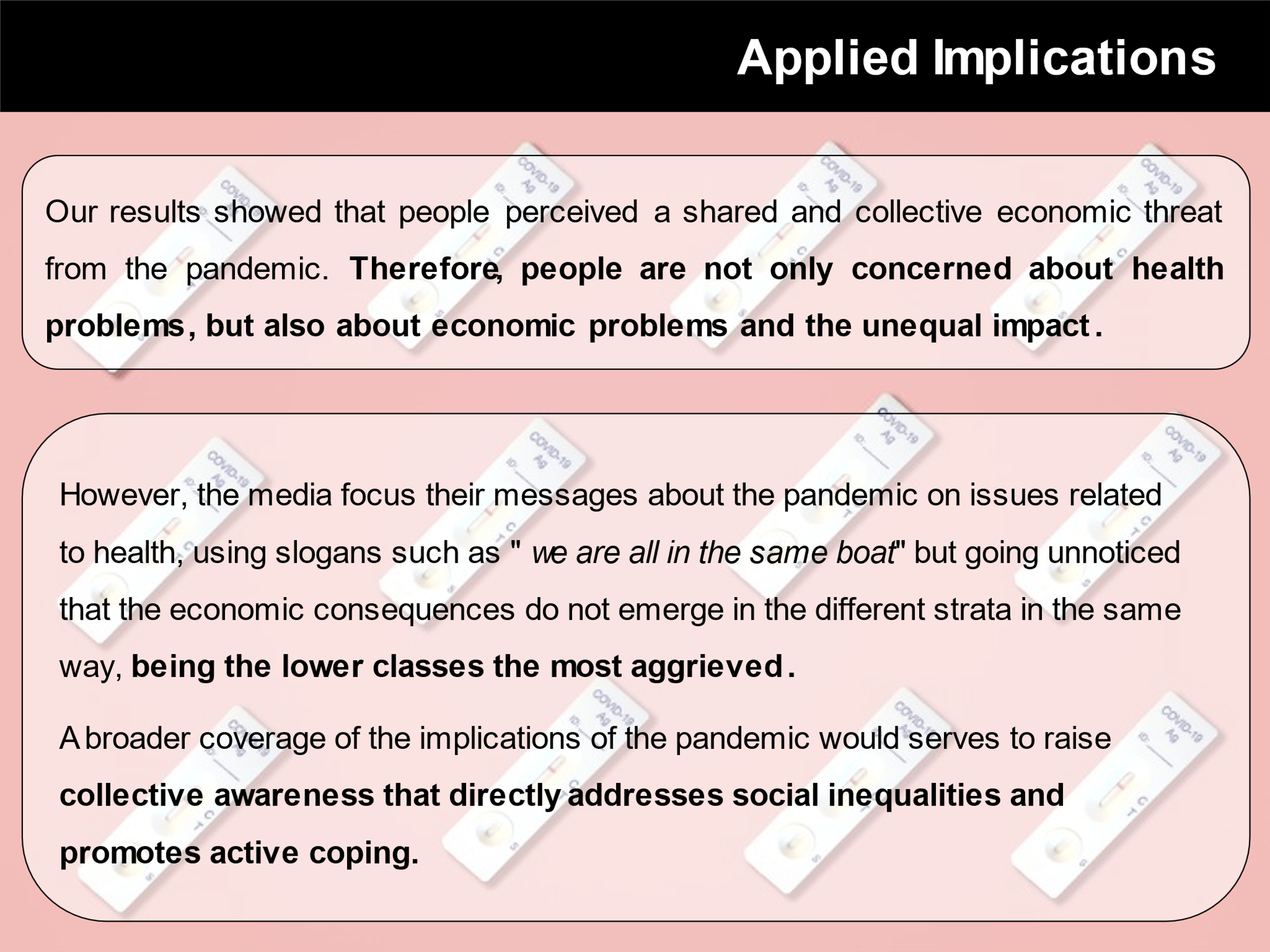


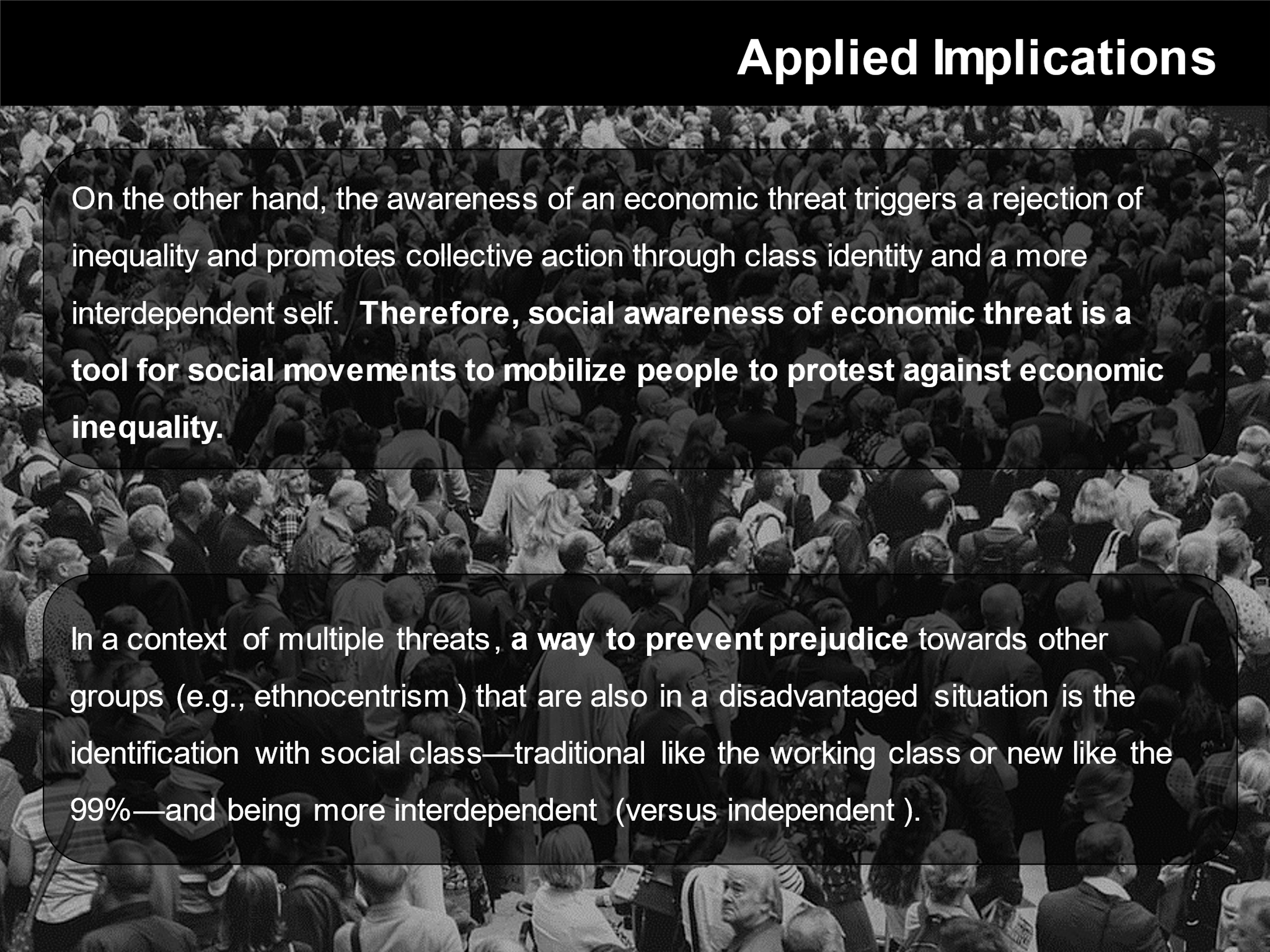


Spanish language:


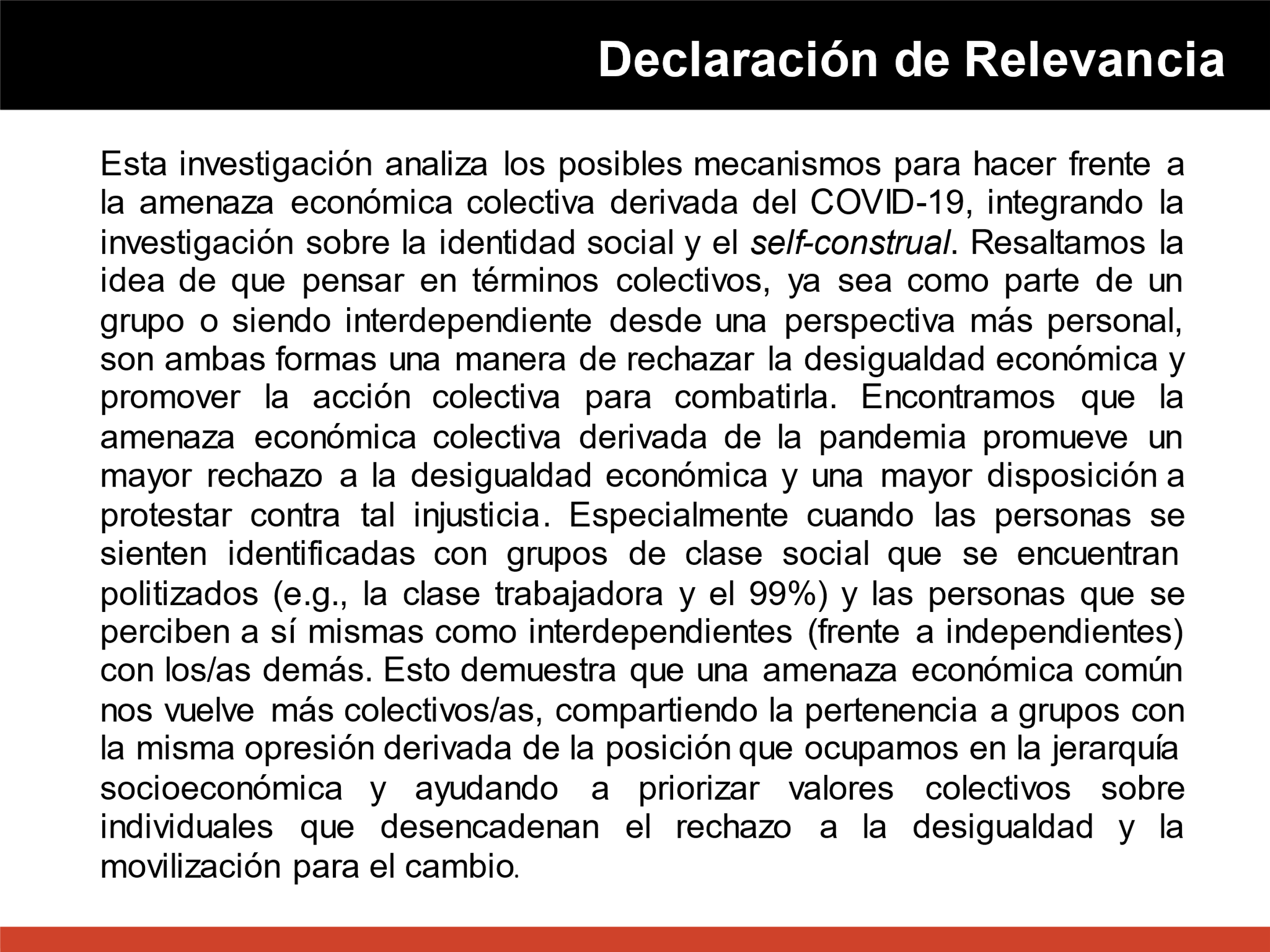


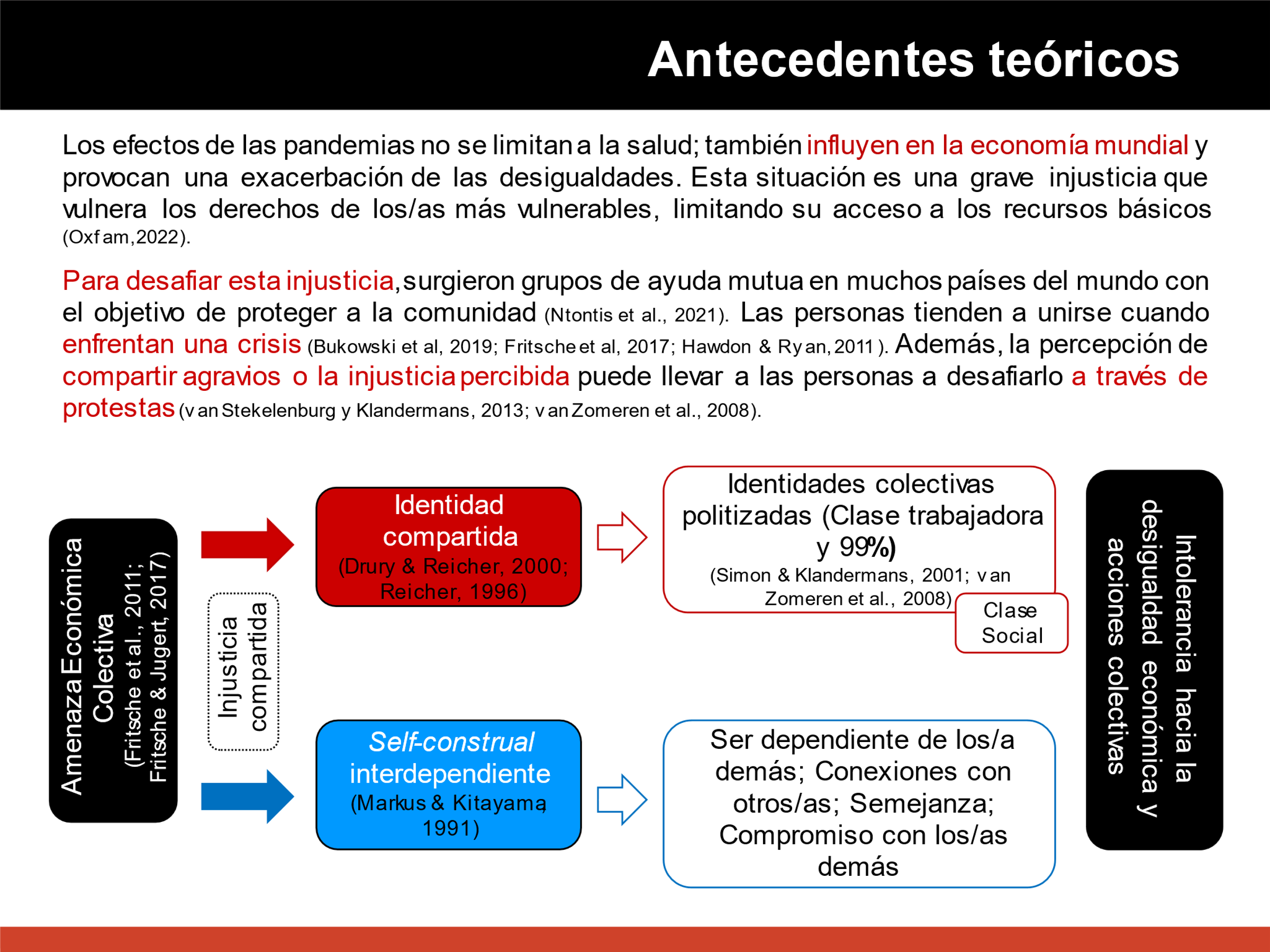


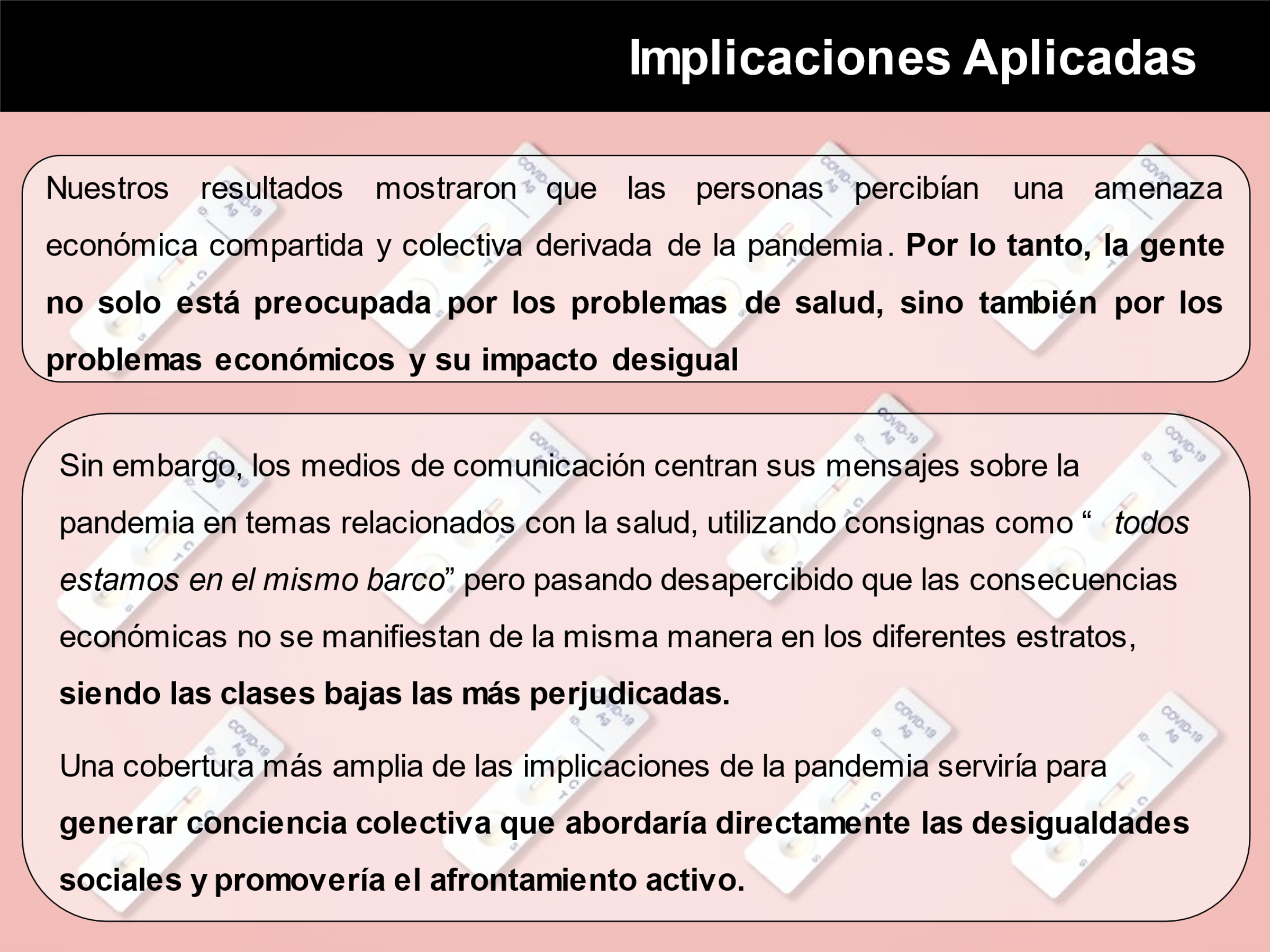


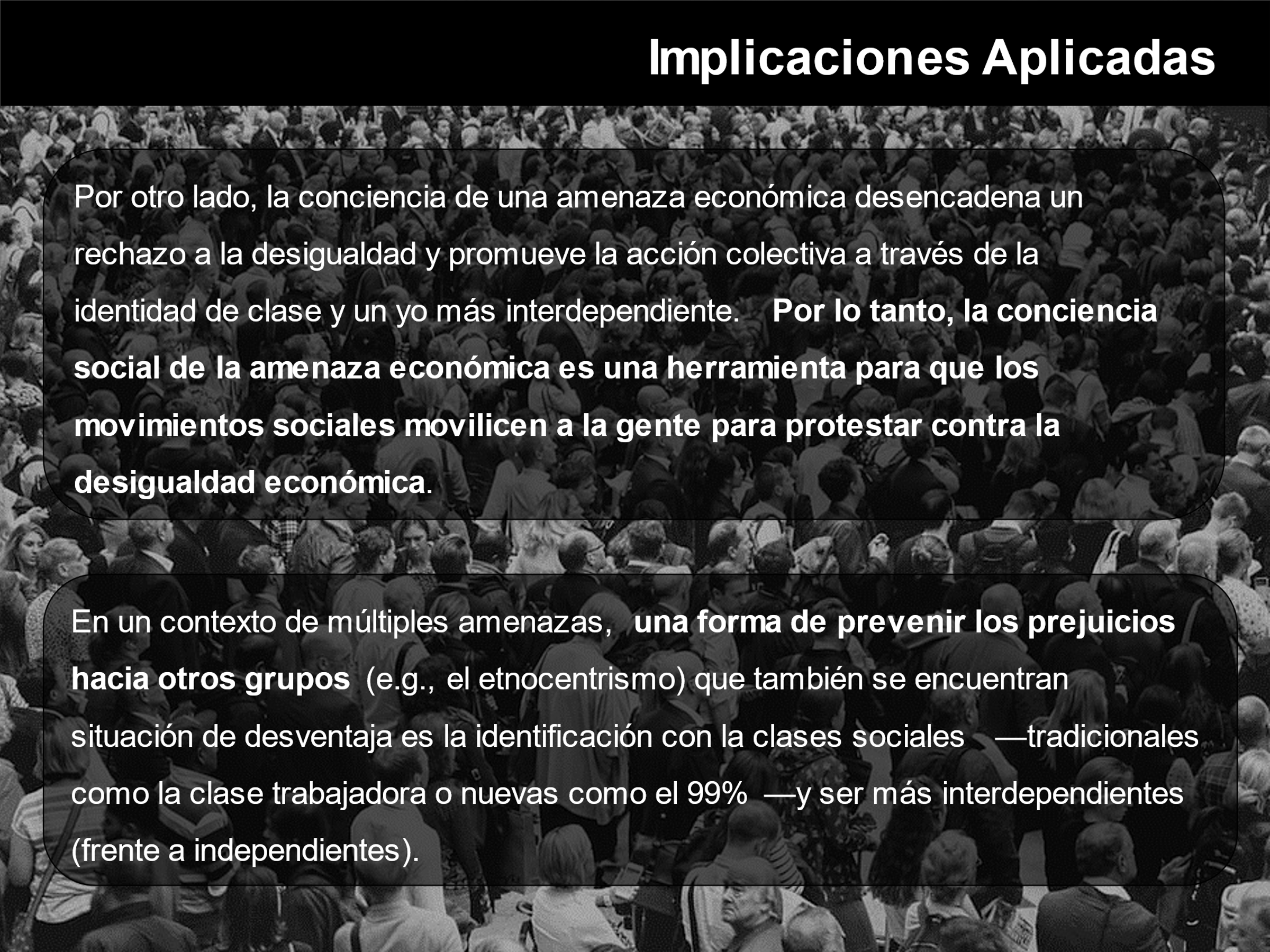


1. MEASURAMENT MATERIALS

**Main variables items**

**Individual Economic Threat**

Think about the current economic situation caused by the coronavirus (COVID-19), that is, how the coronavirus influences people when it comes to coping with their own expenses or their economic/financial position. Please indicate your response to the following statements

Please indicate how you feel about your current financial situation. Please indicate your answer on a scale of 1 to 5, where 1 is "Not at all" and 5 is "Very/Totally"

- How much uncertainty do you feel about your financial situation?
- Do you feel at risk because of your economic situation?
- Do you feel threatened by your economic situation?
- How concerned are you about your financial situation?
- How often do you think about your financial situation?

**Collective Economic Threat**

Now you will have to indicate how you feel about the economic situation in Spain. Think about your family environment, friends, neighbors and acquaintances. Please indicate your answer on a scale of 1 to 5, where 1 is "Not at all" and 5 is "Very/Totally"

- How much uncertainty do you feel about the economic situation in Spain?
- How concerned are you about the economic situation in Spain?
- How often do you think about the economic situation in Spain?

**Group identification**

Next, we will ask you a series of questions about different social groups (99%, working class). Before proceeding to complete them, please read carefully the following information about each of the groups:

- The term 99% vindicates the majority of the world's population (the 99%) against a very small percentage (1%) that owns half of the planet's wealth (if wealth were a cake divided in two, the 1% richest gets one half while the other corresponds to 99% of the world's inhabitants)
- The term working class designates the group of workers who work for a salary in opposition to the ruling class that owns the majority of the property of economic resources.

In relation to the above information, indicate the degree to which you agree or disagree with the following statements. Please indicate your answer on a scale of 1 to 7 to what extent you feel identified with the group in each column, where 1 is "totally disagree" and 7 is "totally agree"

- I identify with the 99%/working class.
- I feel connected to the 99%/working class.
- I feel in solidarity with the 99%/working class.
- I feel committed to the 99%/working class.
- I often think about the fact that I am part of the 99%/working class.
- It is an important part of my identity to be part of the 99%/working class.
- An important part of how I see myself is being part of the 99%/working class.

**Self-Construal Scale**

Below are some statements that someone might use to try to describe you. Some of the statements will probably not describe you well, while others will describe you better. Please check the corresponding number to indicate how well or poorly the phrase describes you. For example, if the statement does not describe you at all, then mark 1. If the statement describes you exactly, then mark 5.

- I prefer to be self-sufficient rather than depend on others
- I try not to depend on other people
- I prefer to turn to other people for help rather than rely solely on myself
- It is important for me to act as an independent person
- I consider that my happiness is independent of the happiness of my friends and family
- It is important for me to be an accepted member of both my family and my group of friends.
- I usually feel a great sense of pride when someone in my family achieves a significant achievement.
- When I think of myself, I often think of my close friends and family.
- I am a unique individual
- Being a unique individual is important to me
- I am a unique person, separate from others
- I enjoy being unique and different from others in many ways
- I would sacrifice my own interest for the benefit of my group
- My relationships with others are more important than my personal achievements
- I will stay in my group if they need me, even when I am not happy with the group
- I stay with my group even despite the difficulties

**Intolerance towards Economic Inequality**

Being 1 "Totally disagree" and 7 "Totally agree", to what extent would you rate the following statements?

- The consequences of economic inequality have been greatly exaggerated
- Economic inequality is causing many of the problems in Spain
- I am very concerned about the degree of inequality that exists in Spain
- Economic inequality is not a problem
- We must do everything possible to reduce the economic inequality that exists in Spain today

**Collective Actions**

Please check below how likely you would be to participate in actions to reduce economic inequality in the current context. Being 1 "Never" and 7 "very often", to what extent would you be willing to carry out the following actions?

- I would vote for political parties whose priority is to establish a special tax on large fortunes to use the proceeds for those who need it most.
- I would promote a campaign to encourage Congress to approve a universal basic income (financial subsidy) for all residents of Spain.
- I would donate money to associations that organize initiatives aimed at financially supporting people in situations of greater vulnerability (e.g., resistance boxes).
- I would join a party, union or political organization against economic inequality.
- I would participate in union activities or political groups that defend that the workers affected by dismissal or ERTES receive 100% of their salary.
- I would participate in peaceful demonstrations that demand the expropriation and nationalization of all private health companies to improve health care for the entire population.
- I would participate in non-violent civil disobedience actions to demand that maximum prices be set for basic necessities (e.g., food, hygiene).
- I would distribute political material (flyers, posters, newspapers) that promoted the distribution of wealth.
- I would boycott products that maintain economic inequality.
- I would sign petitions in favor of economic redistribution.
- Would be active in movements against economic inequality

**Exploratory variables items**

**Health Individual Threat**

Now, think about the current health situation caused by the coronavirus (COVID-19), that is, how the coronavirus influences people's health (contagion, disease...). Please indicate how you feel about your current health. Please indicate your answer on a scale of 1 to 5, where 1 is "Not at all" and 5 is "Very/Totally"

- How much uncertainty do you feel about your health?
- Do you feel your health is at risk?
- Do you feel threatened by your health?
- How concerned are you about your health?
- How often do you think about your health?

**Group identification**

Next, we will ask you a series of questions about different social groups (humanity). Before proceeding to complete them, please read carefully the following information about each of the groups:

- The term humanity comes from a Latin word related to the nature of the human race. It serves to mention the group of human beings that inhabit the planet.

**Collective Health Threat**

Now, you will have to indicate how you feel about the current health situation in Spain. Think about your family environment, friends, neighbors and acquaintances. Please indicate your answer on a scale of 1 to 5, where 1 is "Not at all" and 5 is "Very/Totally"

- How much uncertainty do you feel about the health situation in Spain?
- How concerned are you about the health situation in Spain?
- How often do you think about the health situation in Spain?

**Social Dominance Orientation**

Being 1 "Totally disagree" and 7 "Totally agree", to what extent would you rate the following statements?

- Some groups of people are worth more than others
- We should do everything possible to level the playing field for the different groups
- Sometimes it is necessary to use force against other groups to get what your group wants.
- If certain groups of people held their ground, we would have fewer problems.
- We would have fewer problems if we treated different groups equally.
- To get ahead in life, it is sometimes necessary to step over other groups of people.
- No one group of people should dominate in society.
- Equality between groups of people should be our ideal.
- All groups of people should have equal opportunities in life.
- Social equality must be increased.
- The higher groups of people should dominate the lower groups.
- It is probably a good thing that certain groups are in a higher position and others in a lower position.
- We must fight to achieve more equal income for all.
- Sometimes some groups of people must stay in their position.
- It would be desirable for all groups to be equal.
- The lower groups should stay in their position

**Economic System Justification**

Next, a series of statements about our society will appear. Please mark the number that you think is most convenient to indicate the degree to which you agree or disagree with each of the following statements. Being 1 "Totally disagree" and 5 "Totally agree", to what extent would you rate the following statements?

- If people work hard, they almost always get what they want.
- Most of the people who do not progress in our society should not blame the system: they are the only ones to blame.
- The gap between social classes reflects differences in the natural order of things.
- The economic position of people is a product of their achievements.
- If someone tries hard enough, they can move up the social ladder.
- There are many people who are poor because they don't like to work.
- It is good to have an economic system that rewards those who make an effort.

**Community Collective Actions**

- I would participate in neighborhood groups that have been formed to help the elderly or the population at risk with purchases.
- I would participate in initiatives aimed at collecting funds (solidarity piggy banks or resistance boxes) to financially support the vulnerable population (people affected by ERTE, in an irregular situation, with minors or dependents in their care, etc.).
- I would participate in solidarity actions (such as clothing collection, food bank, etc.) aimed at providing resources for people with economic difficulties.

**Collective Efficacy**

Next, we are going to ask you to remember and try to think about the thoughts you had during the first wave of the pandemic (in confinement: between the months of March and May) and compare them with what you think today, during the second wave of the pandemic. Show your level of agreement or disagreement with each of the following statements, from 1, which is totally disagree, to 7, which is totally agree.

Before:

- Together, citizens, we are stronger
- We citizens can achieve things collectively that cannot be achieved individually
- No one should think that you cannot count on us, the citizens

After:

- Together, citizens, we are stronger
- We citizens can achieve things collectively that cannot be achieved individually
- No one should think that you cannot count on us, the citizens

**Emotions**

Next, we ask you to remember and try to think about how you felt during the first wave of the pandemic (in lockdown: between the months of March and May) and compare it with how you feel today, during the second wave. of the pandemic. Show your level of agreement or disagreement with each of the following statements, from 1, which is totally disagree, to 7, which is totally agree.

Before:

- Illusion, that things could change
- Hope, that as a society we would consider a change of course
- Indignation, before the impact of the pandemic because the pandemic exacerbates economic inequality

After:

- Illusion, that things could change
- Hope, that as a society we would consider a change of course
- Indignation, before the impact of the pandemic because the pandemic exacerbates economic inequality
