# Supplementary material for: Fighting inequalities in times of pandemic: The role of politicized identities and interdependent self‐construal in coping with economic threat
Source: J Community Appl Soc Psychol. 2022 Jun 13:10.1002/casp.2632. Online ahead of print. doi: 10.1002/casp.2632 (PMC9349427; doi:10.1002/casp.2632)
Supplement: Supplementary file 2 — Data S2 Supporting information. [file CASP-9999-0-s001.docx]

**Supplementary Materials**

**pertaining to**

**United in times of pandemic: The role of the politicized identities (against inequality) and interdependent self-construal in coping with economic threat**

This section contains the supplementary material in addition to the manuscript. Specifically, measures, hypotheses and analyses of Studies 1-3 that have been discarded to facilitate understanding from the original manuscript for being inconsistent.

Study 1: Measures (e.g., health threat perception; humanity identification; orientation to social dominance; justification to economic system) and descriptive statistics and correlation.

Study 2-3: Measures (e.g., collective efficacy; emotions; Community collective actions), hypothesis discarded and parallel mediation analyses.

DESCRIPTION OF ADDITIONAL MEASURES AND RESULTS

**Study 1**

**Measures**

Here we discuss measures that were included in the first study that are not described in the main text

**Health threat perception.** We translated and adapted the Financial Threat Scale (FTS) (Marjanovic et al., 2013) to the context of the health threat caused by the coronavirus pandemic. The scale is made up of five items with a Likert scale from 1 to 5. We measured health threat perception. On the one hand, we measured the personal threat with five items (e.g., “How much uncertainty do you feel about your health?*”,* α = .88), and on the other hand, the collective threat with three items (e.g., “How worried are you about the health situation in Spain?”, α = .84).

**Humanity identification.** First, we defined humanity identity “The term humanity comes from a Latin word related to the nature of the human race. It serves to mention the set of human beings that inhabit the planet”. After, we measured, with a Likert scale (1 = Not at all; 7 = Very much) to what extent did participants identify with humanity (α = .89). We measured humanity identification with the centrality and solidarity items of Leach et al., (2008) scale adapted to humanity identity. Seven items made up the measured; three captured the idea of centrality (e.g., “The fact of being part of humanity is an important part of my identity”), and three more captured the idea of solidarity (e.g., “I feel a bond with humanity”). In addition, we included a general item (e.g., “I identify with (in-group)”).

**Orientation to social dominance.** We used the Social Dominance Orientation scale (SDO) (Pratto et al., 1994) translated into Spanish. The scale consisted of sixteen items (Alpha = .82) formed by two main components; Group dominance (α = .72) (e.g., “*The value of some groups of people is greater than that of others*”) and Opposition to equality (Alpha = .84) (e.g., “We should do our best to equalize the conditions for different groups”), with a Likert scale (1 = Totally disagree; 7 = Totally agree).

**Justification of the economic system.** To measure the general ideological tendency to legitimize economic inequality we used a reduced version of the original Economic System Justification scale (ESJ, Jost & Thompson, 2000) adapted and validated into Spanish (Jaume et al., 2012). The scale consisted of seven items (α = .79) (e.g., “If people work hard, they almost always get what they want”) with a Likert scale (1 = Totally disagree; 7 = Totally agree).

**Results**

A summary of the descriptive statistics and correlation between the variables of our Study 1 is presented in Table 1.

**Table 1.** Descriptive statistics and bivariate correlations between the variables measured in Study 1.

|  | CET | IET | CHT | IHT | WC Id. | 99% Id. | H id. | Inter S-C | Intol EI | Coll actions | OSD | JES |
| --- | --- | --- | --- | --- | --- | --- | --- | --- | --- | --- | --- | --- |
| CET | 4.15(0.81) | .21** | .47** | .29** | .31** | .17** | .01 | .16** | .21** | .16** | -.07 | .01 |
| IET |  | 3.03(1.07) | .10 | .09 | .01 | .01 | -.08 | -.07 | .04 | .02 | .12* | .07 |
| CHT |  |  | 3.97(0.84) | .46** | .25** | .14* | .12* | .10* | .17** | .19** | -.07 | .01 |
| IHT |  |  |  | 2.85(0.93) | .19** | .12* | .10 | .12* | -.02 | .01 | .08 | .17** |
| WC Id. |  |  |  |  | 5.97(1.17) | .25** | .21** | .09 | .32** | .31** | -.20** | -.14* |
| 99% Id. |  |  |  |  |  | 5.09(1.79) | .16** | .01 | .23** | .26** | -.11* | -.09 |
| H id. |  |  |  |  |  |  | 6.03(1.16) | .20 | .07 | .11* | -.21** | -.03 |
| Inter S-C |  |  |  |  |  |  |  | 3.12(0.47) | .14** | .18** | -.14** | -.18** |
| Intol EC |  |  |  |  |  |  |  |  | 5.90(1.00) | .53** | -.54** | -.39** |
| Coll actions |  |  |  |  |  |  |  |  |  | 4.88(1.40) | -.45** | -.42** |
| OSD |  |  |  |  |  |  |  |  |  |  | 2.07(0.77) | .44** |
| JES |  |  |  |  |  |  |  |  |  |  |  | 2.40(0.74) |

*Note*: * *p* ≤ 0.05; ***p* ≤ 0.01; C*ET*., Collective Economic Threat; *IET*., Individual Economic Threat; *CHT*., Collective Health Threat; *IHT*., Individual Health Threat; *WC Id*., Working class identification; *99% id*., 99% identification; *H id*., Humanity identification; *Inter. S-C*., Interdependent Self-Construal; *Intol EC*., Intolerance towards Economic Inequality; *Coll actions*., Collective actions; *OSD*., Orientation to Social Dominance; *JES*., Justification of Economic System. Diagonal shows mean of the participants’ score in the scale and standard deviation in brackets.

**Study 2 & 3**

**Pre-registered hypothesis discarded:**

Hypothesis 3. We predicted two indirect effects in parallel. The relation between the collective economic threat and the community collective actions are mediated, in the one hand, by the levels of identification with the identity of the working class, and on the other hand, by the interdependent self-construal. In the sense that the perception of economic collective threat because of COVID-19 will lead participants to identity more strongly with the working class and to show more interdependent self-construal, and both will lead to increase the willingness to participate in community collective actions.

Hypothesis 4, 5 y 6. We predicted a significant mean difference on the participant’s perception of illusion, hope and indignation between the first and second waves of pandemic. We expected that the emotions of illusion and hope that the participants remember having felt in the first wave of the pandemic will be significantly greater that the emotions of illusion and hope that participants felt in the second wave of the pandemic. Also, the emotions of indignation that the participants remember having felt in the first wave of the pandemic will be significantly lower than the emotions of indignation that participants felt in the second one.

Hypothesis 7. We predicted a significant mean difference of collective efficacy between the first and second waves of pandemic. We expected that the collective efficacy that the participants remember having perceived in the first wave of the pandemic will be significantly greater than the collective efficacy that the participants perceived in the second one.

***Measures***

In the same way as in Study 1, some measures and results did not appear in the main text. Here we discuss measures that were included in the Study 2-3 that are not described in the main text

**Collective efficacy.** We used four items translated into Spanish (Stolleberg et al., 2015) (e.g., “Together, the citizens, we are stronger”). We measured in two different ways. First, we asked participants to recall the first wave of the pandemic (Study 2: α = .76; Study 3: α = .82) and, second, we asked how they perceive it in the current wave (Study 2: α = .82; Study 3: α = .84).

**Emotions.** In addition, we included three items to measure hope (“Hope that as a society a change of course will be considered”) (Study 2: r = .18 p = .005; Study 3: r = .211, p < .001), illusion (“Illusion that things could change”) (Study 2: *r* = .147, *p* = .021; Study 3: *r* = .133, *p* < .01) and indignation (“Indignation and the impact of the pandemic because the pandemic exacerbates economic inequality”) (Study 2: *r* = .645, *p* < .001; Study 3: *r* = .618, *p* < .001). Following the same logic than in the previous measure, we asked participants to recall the emotions that they experienced during the first wave of pandemic, and second one**.**

**Community collective actions**. We also measured community collective actions with four items (e.g. It would participate in neighbourhood groups that have been formed to help with purchases for the elderly and the population at risk; Study 2: α = .88; Study 3: α = .87).

**Results**

Some results do not appear in the main manuscript due to facilitate of understanding or inconsistent results.

***Social identity and self-construal as mediators between collective economic threat and community collective actions***

Then, we carried out parallel mediation analyses with PROCESS (model 4; Hayes, 2013) to test the role of 99% identity (M1), working class identity (M2) and the interdependent self-construal (M3) as potential mediators of the relationships between collective economic threat (X) and the community collective action (Y). We used 5,000 bootstrap samples to estimate bias-corrected standard errors and 95% percentile confidence intervals for the indirect effects. We included the covariates: sex, age, political orientation and subjective economic status.

In Study 2, neither the total effect of collective economic threat on community collective actions (*b* = 0.14, 95% CI [-0.09, 0.36]; *p* = .237), nor the direct effect (*b* = 0.06, 95% CI [-0.17 0.29]; *p* = .613) were significant. The indirect effects via the working class identity (*b* = 0.03, 95% CI [-0.01,0.10]), and 99% identity (*b* = 0.04, 95% CI [-.01,0.04]) were not significant. Otherwise, the indirect effect via interdependent self-construal was significant (*b* = 0.04, 95% CI [0.01,0.10]).

In Study 3, we found that the total effect of collective economic threat on community collective actions was significant (*b* = 0.24, 95% CI [0.08, 0.40]; *p* = .003), also the direct effect (*b* = 0.22, 95% CI [0.05, 0.38]; *p* = .009). Neither the indirect effects via the 99% identity (*b* = -0.01, 95% CI [-0.02,0.24]), nor the indirect effect via interdependent self-construal (*b* = -0.01, 95% CI [-0.02,0.02]) were significant. Otherwise, the indirect effect via working class identity was significant (*b* = 0.03, 95% CI [0.01,0.08]).

***Differences on the perception of emotions and collective efficacy between the first and second wave of pandemic***

In Study 2, we conducted a t-test to explore possible differences on the perception of emotions (illusion, hope and indignation) as well as on collective efficacy over time. The results showed significant differences between pandemic waves on illusion, *t*(244) = 12.764, *p* < .001, *d* = 1.07; hope, *t*(244) = 13.74, *p* < .001, *d* = 1.12; and indignation, *t*(244) = -7.84, *p* < .001, *d* = .43). In such a way that participants perceived in the first pandemic wave more illusion (*M* = 5.20; *SD* = 1.81) and hope (*M* = 4.93; *SD* = 1.78) but lower indignation (*M* = 5.31; *SD* = 1.72) that in the second pandemic wave (illusion: *M* = 3.28; *SD* = 1.79; hope: *M* = 2.97; *SD* = 1.71; indignation: *M* = 5.98; *SD* = 1.36). However, differences on perceived collective efficacy between the first (*M* = 5.32; *SD* = 1.30) and the second pandemic waves (*M* = 5.15; *SD* = 1.50) were not significant, *t*(244) = 1.907, *p* = .058, *d* = .121).

The results of Study 3 showed significant differences between pandemic waves on illusion, *t*(405) = 17.28, *p* < .001, *d* = 1.116; hope, *t*(405) = 18.68, *p* < .001, *d* = 1.17; indignation, *t*(404) = -8.52, *p* < .001, *d* = 0.38; and collective efficacy *t*(404) = 5.76, *p* < .001, *d* = 0.30. In such a way that participants perceived in the first pandemic wave more illusion (*M* = 5.27; *SD* = 1.67) and hope (*M* = 5.00; *SD* = 1.80) but less indignation (*M* = 5.36; *SD* = 1.64) that in the second pandemic wave (illusion: *M* = 3.44; *SD* = 1.61; hope: *M* = 2.99; *SD* = 1.64; indignation: *M* = 5.93; *SD* = 1.36). Finally, the means of perceived collective efficacy in the first wave (*M* = 5.45; *SD* = 1.25), was higher than in the second wave (*M* = 5.04; *SD* = 1.52), *t*(404) = 5.761, *p* < .001, *d* = 0.295).

.
